# ApoE4-specific Misfolded Intermediate Identified by Molecular Dynamics Simulations

**DOI:** 10.1371/journal.pcbi.1004359

**Published:** 2015-10-27

**Authors:** Benfeard Williams II, Marino Convertino, Jhuma Das, Nikolay V. Dokholyan

**Affiliations:** Biochemistry and Biophysics Department, University of North Carolina, Chapel Hill, Chapel Hill, North Carolina, United States of America; Tel Aviv University, ISRAEL

## Abstract

The increased risk of developing Alzheimer’s disease (AD) is associated with the *APOE* gene, which encodes for three variants of Apolipoprotein E, namely E2, E3, E4, differing only by two amino acids at positions 112 and 158. ApoE4 is known to be the strongest risk factor for AD onset, while ApoE3 and ApoE2 are considered to be the AD-neutral and AD-protective isoforms, respectively. It has been hypothesized that the ApoE isoforms may contribute to the development of AD by modifying the homeostasis of ApoE physiological partners and AD-related proteins in an isoform-specific fashion. Here we find that, despite the high sequence similarity among the three ApoE variants, only ApoE4 exhibits a misfolded intermediate state characterized by isoform-specific domain-domain interactions in molecular dynamics simulations. The existence of an ApoE4-specific intermediate state can contribute to the onset of AD by altering multiple cellular pathways involved in ApoE-dependent lipid transport efficiency or in AD-related protein aggregation and clearance. We present what we believe to be the first structural model of an ApoE4 misfolded intermediate state, which may serve to elucidate the molecular mechanism underlying the role of ApoE4 in AD pathogenesis. The knowledge of the structure for the ApoE4 folding intermediate provides a new platform for the rational design of alternative therapeutic strategies to fight AD.

## Author Summary

ApoE is a lipid transporter protein associated with the onset of Alzheimer’s disease. Among the three human variants, ApoE4 isoform represents the strongest genetic risk factor for the onset of Alzheimer’s disease. We identify an ApoE4-specific misfolded intermediate state that may alter multiple cellular pathways involved in cellular homeostasis of lipids and AD-related proteins. The presented structural model of the ApoE4-specific intermediate state may serve to elucidate the molecular mechanisms underlying the physiopathological functions of ApoE isoforms, and it can pave the way for the development of alternative pharmacological strategies in the treatment of Alzheimer’s disease.

This is a *PLOS Computational Biology* Methods paper

## Introduction

ApoE is a polymorphic lipid binding protein found in the human liver and brain [1,2], that has been shown to play a role in neuronal repair and maintenance [3]. The three common ApoE isoforms in humans are ApoE2, ApoE3 and ApoE4 [4]. They differ by only two amino acids, cysteine and arginine, at positions 112 and 158 in the N-terminal domain (see Table 1 and S1 Table), but have noticeable differences in their biochemical function such as the formation of lipoprotein bundles [3–7]. Importantly, ApoE4 has been shown to be the strongest genetic risk factor for AD. Indeed, it has been shown that the risk for AD increases from 20% to 47% to 91% in non-carrier (*ApoE4 -/-*), heterozygous (*ApoE4 +/-)*, and homozygous (*ApoE4 +/+)* subjects, respectively. Concurrently, the age of AD onset decreases by over 15 years in homozygous individuals [8–12]. In contrast, ApoE2 and ApoE3 have been found to be respectively protective and neutral in terms of propensity to develop AD [9–12]. Despite these strong correlations, the relationship between the structure of the three ApoE isoforms and their contribution to AD etiology is still unknown. Furthermore, there is strong evidence that these structural/dynamical differences between ApoE isoforms contribute to differences in disease onset and progression [13].

**Table 1:** ApoE isoform-specific mutations.

ApoE isoform	Residue 112	Residue 158
ApoE2	Cys	Cys
ApoE3	Cys	Arg
ApoE4	Arg	Arg

The structure of ApoE was determined in X-ray crystallography [14,15] and nuclear magnetic resonance (NMR) [16]: ApoE consists of an N-terminal domain with a four-helix bundle, a hinge region and a flexible C-terminal domain (S1A Fig) The N-terminal and the C-terminal domains contain the lipoprotein receptor’s binding sequence and the lipids’ binding site, respectively [14,17]. Recent studies have revealed that ApoE undergoes structural rearrangements upon binding events [18], and its accessibility to intermediate states differs for each isoform [5].

The ApoE4 mutation leads to decreased thermal stability of the protein that may allow access to a stable intermediate conformation promoting pathological consequences [5]. Indeed, it has been suggested that this misfolded intermediate state of ApoE4 can potentially be responsible for ApoE isoform-specific effects on AD-related proteins, such as amyloid beta (Aβ) peptide and tau protein [18]. Indeed, it has been shown that ApoE isoforms differently affect the oligomerization rates of Aβ peptide, and ApoE4 specifically stabilizes Aβ peptide intermediate states [19–21]. Additionally, recent studies show that, in the presence of ApoE4, tau protein is hyperphosphorylated, which then lead to formation of toxic intracellular neurofibrillary tangles [22–25]. Therefore, the formation of a putative ApoE4-specific misfolded intermediate state can potentially underlie the higher risk of AD associated with this isoform [5]. However, the tendency of these proteins to form oligomers in solution [26] presents a major complication in the experimental investigation of their folding mechanism, and in the identification of the structural features characterizing each ApoE isoform [27]. Here, we explore the conformational landscape of the three ApoE isoforms, using discrete molecular dynamics (DMD, [28–30]), in order to investigate the structural determinants that distinguish each isoform. Our goal is to elucidate the plausible isoform-specific structural features that could underlie the physiopathological function of each ApoE variant. We observe several intermediate states for each ApoE isoform in our simulations. Specifically, we identify an ApoE4-specific misfolded intermediate state characterized by a unique group of contacts that mediate the interaction between the N-terminal and C-terminal domains of the protein (domain-domain interaction). This misfolded intermediate state can potentially play a pivotal role in AD pathogenesis, by altering multiple ApoE functional pathways, such as lipid transport efficiency, Aβ peptide clearance and aggregation of Aβ peptide, and/or tau protein hyperphosphorylation.

## Results

### Thermodynamic stability of ApoE isoforms

**Fig 1:**
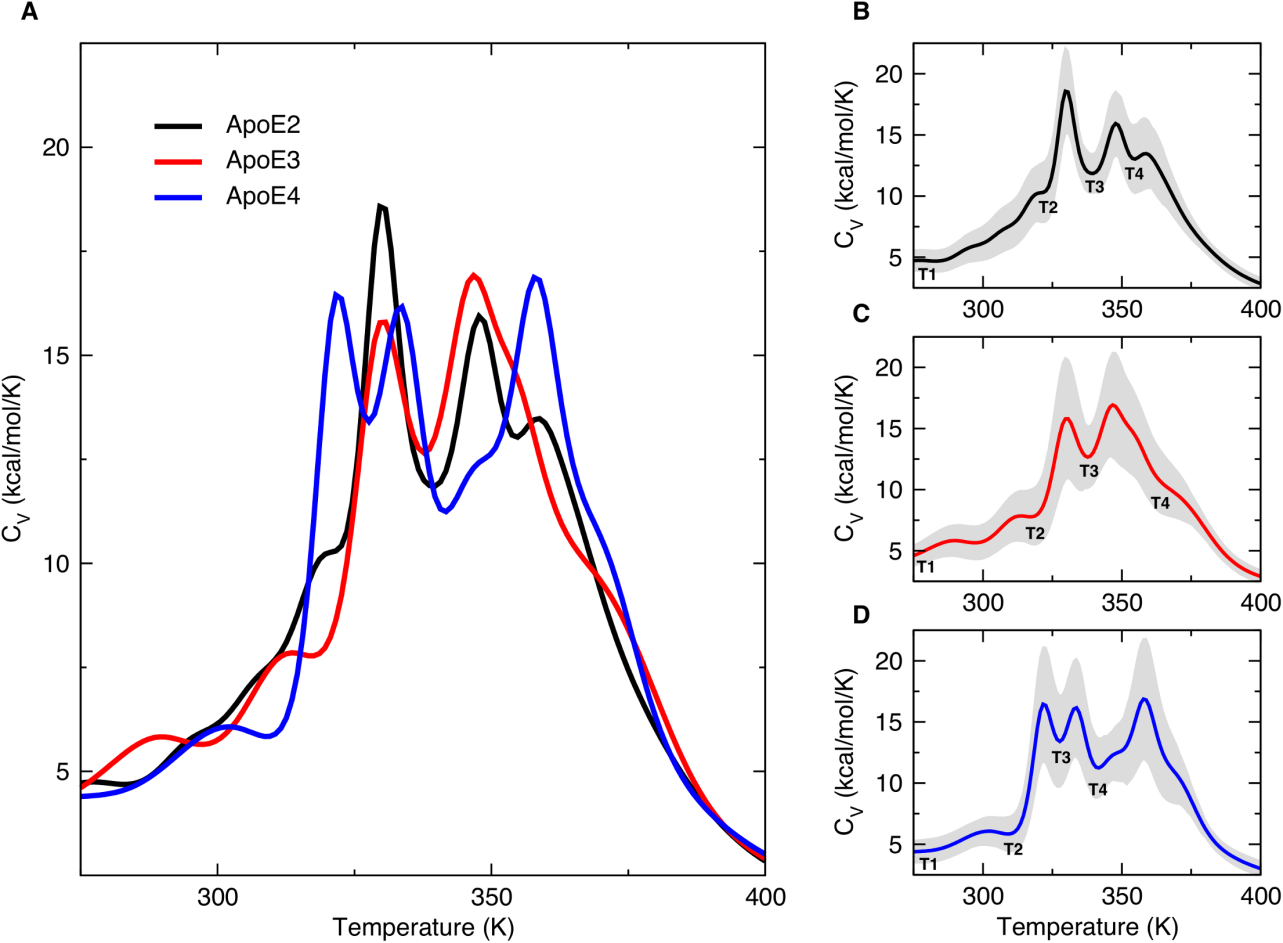
Heat capacity curves of ApoE isoforms. (A) The heat capacity (Cv) curves computed using WHAM on REX/DMD trajectories for ApoE2 (black), ApoE3 (red) and ApoE4 (blue) in the range of 275 to 400 K show intermediates states that appear at different temperatures for each isoform. The position of the first peak (*i*.*e*., unfolding of the hydrophobic core of the protein) suggests that ApoE4 is less thermally stable than ApoE2 and ApoE3. (B-D) Cv curves of individual ApoE isoforms including the error bars (shaded grey area). The shaded grey area in panels B-D represents the statistical uncertainty (*i*.*e*., the square root of the variance of the specific heat) in the WHAM estimation of heat capacity. Local minima in the curves at temperatures T1, T2, T3, and T4 represent different conformational states of the protein for each ApoE variant.

To evaluate the thermodynamic stability of the three isoforms of ApoE, we compute the heat capacity of each variant (Fig 1) by applying the Weighted Histogram Analysis Method (WHAM) [31] to replica exchange DMD (REX/DMD) simulation trajectories as described in the Simulation settings section in Methods. In all three ApoE isoforms’ specific heat curves, we observe several peaks suggesting the existence of multiple intermediate states for each of them (Fig 1B–1D). To estimate the relative thermal stability of the three ApoE isoforms, we refer to the temperature of the first peak in the specific heat plots, corresponding to the temperature at which the N-terminal domain of the three ApoE variants loses its hydrophobic core packing, as described below. A left shift of this peak in the specific heat plots indicates destabilization of the protein structure, while a right shift implies its stabilization. The temperature of the first peak in ApoE4’s specific heat curve (322 K) is lower than that of ApoE2 (330 K) and ApoE3 (331 K). Therefore, we infer that ApoE2 and ApoE3 show very similar thermal stability in simulation, while ApoE4 is characterized by a lower stability with respect to these two isoforms, in agreement with previously reported results in the literature [32,33] (Fig 1A). These data are in agreement with ΔΔG values estimated by an independent computational approach, Eris (Simulation settings section in Methods), in which the free energy is estimated as a weighted sum of van der Waals forces, solvation, and hydrogen bond energy functions [34,35].

To determine if the differences in the specific heat of the three ApoE isoforms are due to the temperature-induced unfolding of different regions of the protein, we monitor the secondary structure content as a function of temperature for every residue (S1B–S11D Fig) and for the entire protein of each ApoE variant (S1E–S1G Fig). Our results suggest that, despite their different thermal stabilities, the three ApoE isoforms undergo temperature-induced unfolding without significant differences in the loss of secondary structure (S1B–S1G Fig) or tertiary structure content, (S2J–S2L Fig) thus, implying a major role of potential structural rearrangements within the domains of the three protein isoforms.

### Unfolding transitions and representative states of ApoE isoforms

**Fig 2:**
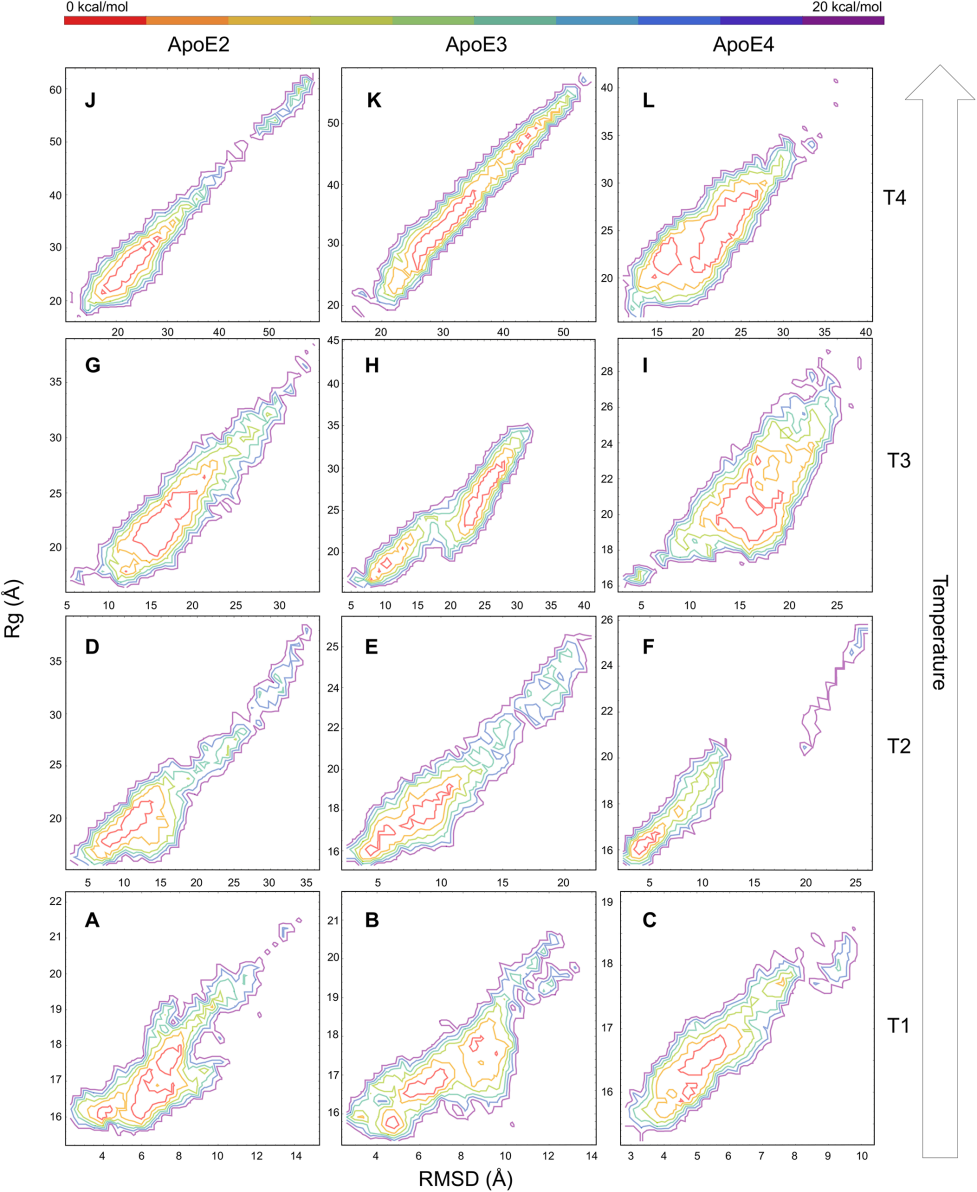
Free energy landscapes of ApoE isoforms. ApoE isoforms’ conformational landscapes derived from PMF as a function of RMSD and Rg of ApoE variants’ N-terminal domains. C-terminal domains are excluded from the analysis to reduce the degeneracy of protein conformational states. (A-C) The free energy landscapes from REX/DMD simulations at T1 (~275 K for all three ApoE isoforms) are isolated in the lowest range of RMSD and Rg suggesting the majority of conformations are close to the native N-terminal domain state. (D-F) At T2 (~321 K, ~318 K, and ~309 K for ApoE2, ApoE3 and ApoE4 respectively) all three variants explore a larger area of the conformational landscape as denoted by the larger RMSD and Rg values. (G-I) At T3 (~340 K, ~338 K, and ~328 K for ApoE2, ApoE3 and ApoE4 respectively) the isoforms transition to their intermediate states. ApoE3 is characterized by both the native and alternate N-terminal domain conformations, while ApoE2 visits only the latter. ApoE4 exhibits a unique, more compact intermediate conformational state as denoted by the smaller range of RMSD and Rg values compared to the two other variants. (J-L) At T4 (~355 K, ~365 K, and ~342 K for ApoE2, ApoE3, and ApoE4, respectively) all three isoforms undergo complete unfolding as inferred by their extended landscapes in the high range of RMSD and Rg values, although ApoE4 also visits the previous conformational states identified at temperature T3. (Note the different scale on x- and y-axes; representative structures are reported in S2 Fig). The color bar represents the relative Helmholtz free energy in kcal/mol.

**Fig 3:**
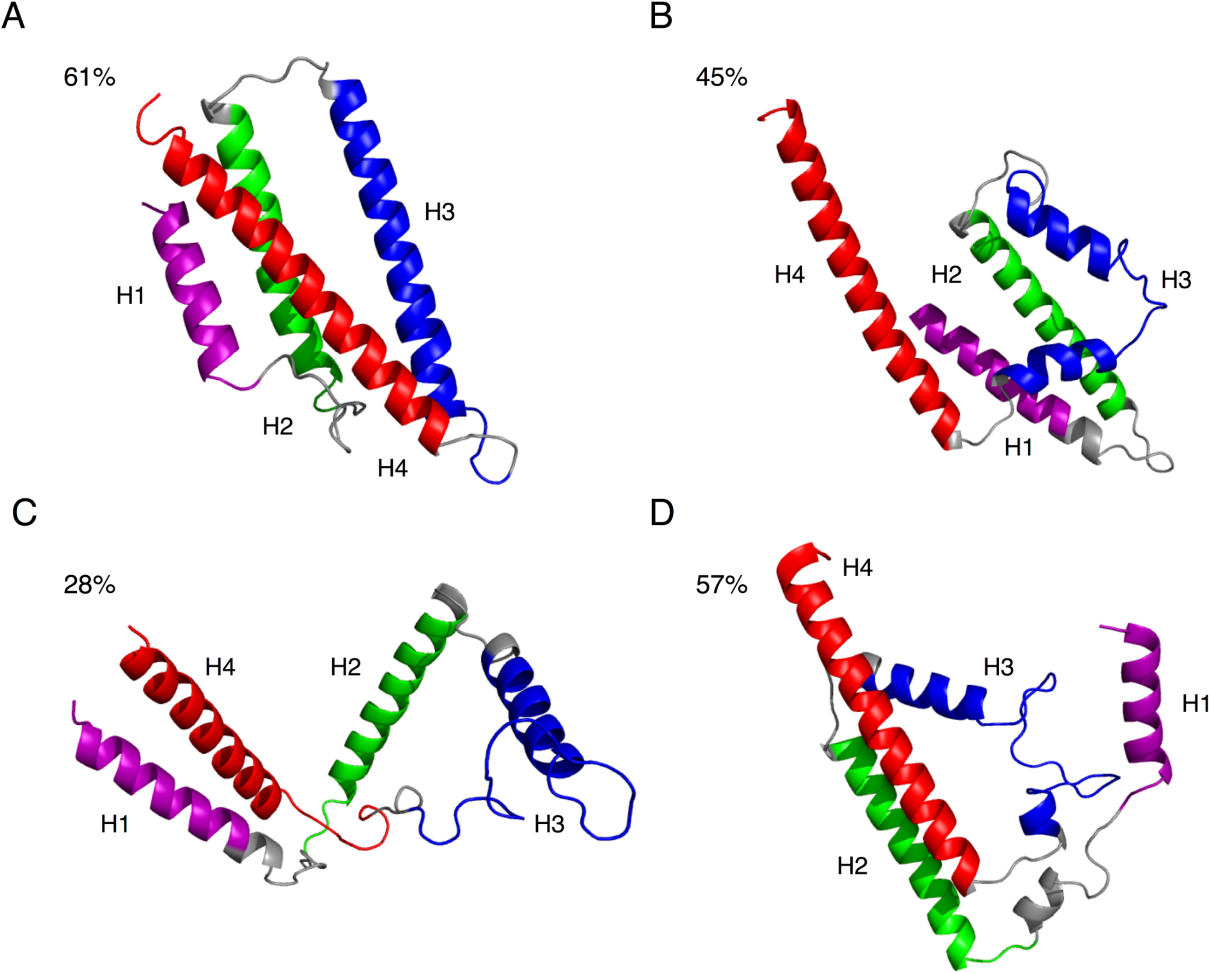
Representative structures of ApoE isoforms. (A) ApoE3 representative structure (*i*.*e*., centroid of the most populated cluster) from clustering analysis of the protein conformations extracted from the free energy basin at T1 (~275 K, see Fig 2B). The same compact, native state of the N-terminal helices is observed in all three ApoE isoforms (see S2A–S2C Fig). At T3 (~340 K, ~338 K, and ~328 K for ApoE2, ApoE3 and ApoE4 respectively) the representative structure of the intermediate state for: (B) ApoE2 exhibits an expanded volume of the N-terminal domain due to an increase of the average inter-helical distances; (C) ApoE3 exhibits a pairing of N-terminal helix–1 and helix–4 which separate from helix–2 and helix–3; (D) ApoE4 exhibits a separation of helix–1 from the other three helices. Such conformation represents the identified isoform-specific misfolded intermediate state (inter-residue contacts shown in S6A Fig) The size of the most populated cluster is reported in each panel. For all structures, helix–1 (H1), helix–2 (H2), helix–3 (H3), and helix–4 (H4) are represented in purple, green, blue, and red, cartoon respectively. The rest of the protein is represented in grey cartoon. (The sequence numbers for helices is reported in S1 Fig).

To discriminate isoform-specific structural features underlying differential thermal stability in ApoE variants, we isolate the isoforms’ representative conformations by calculating the Potential of Mean Force (PMF) (See Methods) for ApoE2, ApoE3 and ApoE4, at different temperatures (defined as T1, T2, T3 and T4, and having different values in each ApoE variant). We use the root mean-square deviation (RMSD) and the radius of gyration (Rg) of the N-terminal domain as collective variables for the PMF calculations. We exclude from the analysis the highly flexible C-terminal domain (see RMSD distribution in S3 Fig) to reduce the degeneracy of protein conformational states in the PMF calculations. In order to identify highly probable ApoE conformations at a given temperature (S2 Fig), we apply an RMSD-based clustering approach (Simulation analyses section in Methods) to the ensemble of conformations isolated from low energy basins in the free energy landscapes (Fig 2). At the lowest temperatures T1 (275 K) and T2 (corresponding to 321 K for ApoE2, 318 K for ApoE3 and 309 K for ApoE4) (Fig 2A–2F), we find compact, native-like N-terminal domain conformations with similar contacts, comparable to the crystallographic structures reported in the Protein Data Bank (S2 Table and S4 and S5 Figs). The N-terminal domain conformations are similar to what is observed in the ApoE3 conformation (Fig 3A and S3 Table), and are characterized by an RMSD within 9 Å and an Rg within 3 Å of the respective starting structures for all three ApoE isoforms (S2A–S2F Fig).

The free energy landscapes at temperatures T3 (corresponding to 340 K for ApoE2, 338 K for ApoE3 and 328 K for ApoE4) represent the conformational states associated with the local minima between the first and second peaks in the heat capacity plots (Fig 2G–2I). At these temperatures, the hydrophobic core of the N-terminal domain is differentially perturbed in the three ApoE isoforms, while this behavior is less apparent at physiological temperatures (S4 Table). Specifically, we observe a decrease in the hydrophobic contacts of ApoE2’s N-terminal domain helices (Fig 3B) and ApoE3’s N-terminal domain separating into two helix pairs (Fig 3C). More importantly, we identify a unique intermediate state for ApoE4 (Discussion section), in which only helix–1 separates from the N-terminal domain helix bundle (Fig 3D). The unfolding of the N-terminal helix–3 is a feature shared by all three isoforms as characterized by the analysis of the secondary structure profiles (S1B–S1D Fig). At temperatures T4 (corresponding to 355 K for ApoE2, 365 K for ApoE3 and 342 K for ApoE4), which describes the final local minima of the specific heat curves (Fig 2J–2L), all three of the ApoE isoforms lose their tertiary structure (S2J–S2L Fig) and undergo complete unfolding.

### Inter-domain interactions of ApoE isoforms

**Fig 4:**
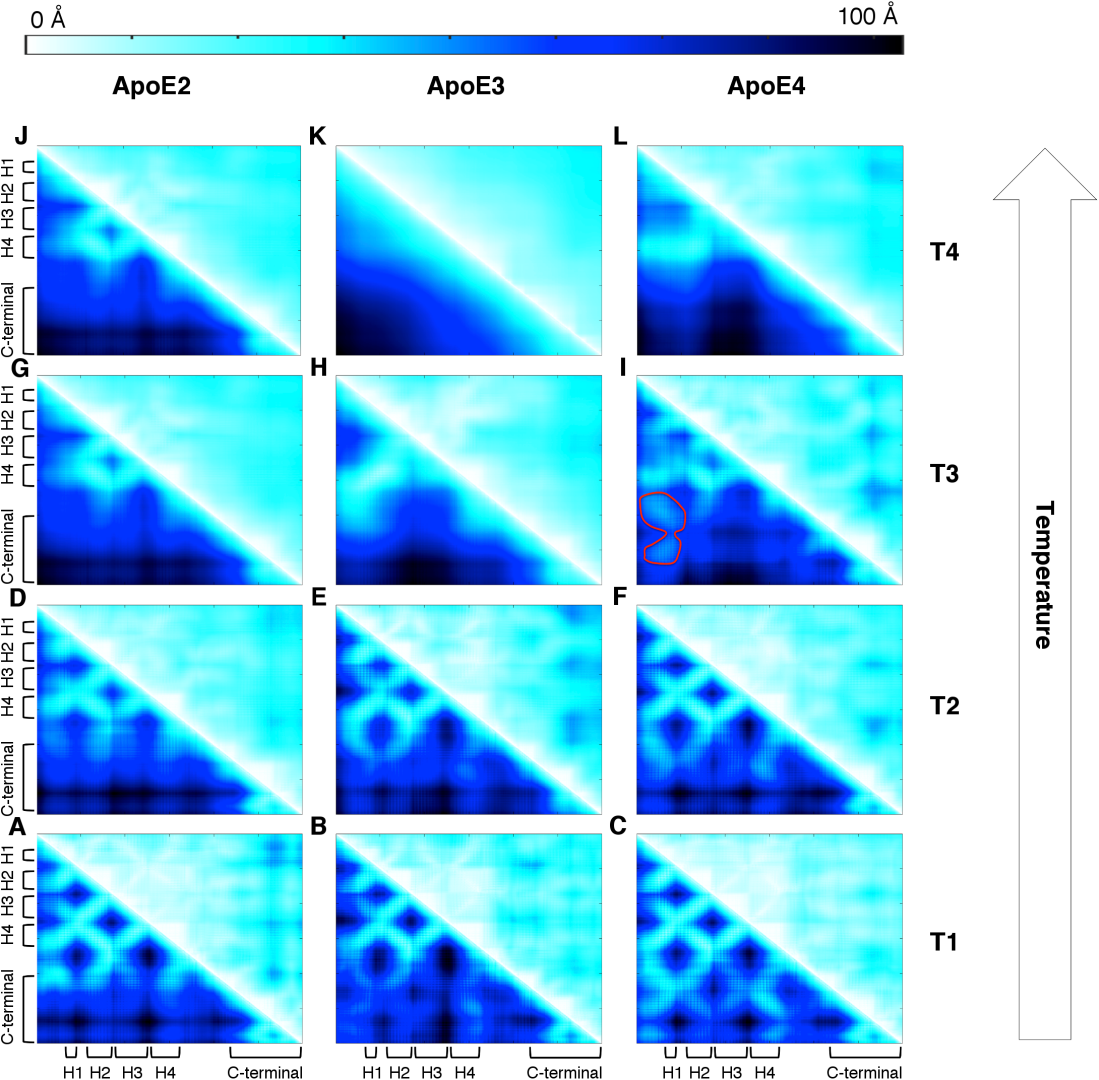
Temperature-dependent pair-wise inter-residue distances of ApoE isoforms. (A-C) At T1 (~275 K for all three ApoE isoforms) and (D-F) at T2 (~321 K, ~318 K, and ~309 K for ApoE2, ApoE3 and ApoE4 respectively), all three isoforms exhibits the highest level of inter-residue contacts observed in the REX/DMD simulations, with ApoE4 having the highest density contacts. (G-I) At T3 (~340 K, ~338 K, and ~328 K for ApoE2, ApoE3 and ApoE4 respectively), all three isoforms exhibit a dramatic decrease in density of inter-residue contacts. ApoE4 displays a unique series of contacts (outlined in red) mediating the domain-domain interaction as discussed in the main text. (J-L) At T4 (~355 K, ~365 K, and ~342 K for ApoE2, ApoE3, and ApoE4, respectively), the majority of inter-residue contacts have been lost besides some transient contacts involving the N-terminal helix–4. The upper and lower triangular matrices represent respectively the average and the standard deviation of the pair-wise inter-residue distance in Å. The color bar represents the distance between the centroid computed over the residues’ side chains in Å.

To identify the physical interactions that characterize the intermediate states of each ApoE isoform, we compute inter-residue distances for each ApoE isoform (Simulation analyses section in Methods). At the lowest temperatures T1 and T2 (Fig 4A–4F), we detect a high number of contacts between the N- and C-terminal domains for each ApoE variant suggesting the existence of compact, native-like N-terminal domain structures. ApoE4 exhibits the highest density of contacts between N- and C-terminal domains at temperatures T1 and T2 (Fig 4C and 4F).

At temperature T3 (the first local minima in the specific heat plot in Fig 1), we observe a lack of contacts between the N- and C-terminal domains in ApoE2 and ApoE3 (Fig 4G and 4H) and a diminished number of contacts in ApoE4 (Fig 4I). Additionally, we observe a decrease in N-terminal intra-domain contacts for all of the three ApoE isoforms indicating the loss of hydrophobic packing in all ApoE variants.

For the highest temperature in our analysis (*i*.*e*., T4), we note that the majority of all contacts are lost (Fig 4J–4L), with the exception of some transient interactions involving the N-terminal helix–4 in ApoE2 and ApoE4, which follows the loss of secondary and tertiary structure observed in other analyses (S1B–S1G and S2J–S2L Figs).

Interestingly, for ApoE4 at temperature T3, we observe a unique series of contacts between residues 26 to 44 in the N-terminal helix–1, residues 196 to 215 in the hinge region, and residues 235 to 243 in the C-terminal domain (Fig 4I outlined in red). These contacts are a representative structural feature of all the ApoE4 conformations throughout the entire free energy landscape. The same contacts are present in the most populated cluster extracted from the ApoE4 free energy basin at temperature T3 (S7 and S8 Figs) indicating the existence of a stable, representative inter-domain interaction between the specified residues that characterize an ApoE4-specific misfolded intermediate state (Fig 3D).

In previous studies Dong and co-workers have suggested that increased inter-domain interaction in the ApoE4 isoform is mediated specifically by the formation of a salt-bridge between R61 and E255 [36,37]. However, we do not observe these two residues in contact when we monitor the distance between them in our REX/DMD simulations of ApoE4 (S9A and S9B Fig). On the other hand, previously reported experimental distances between residues mediating the formation of a misfolded ApoE4 intermediate state, and associated with R61-E255 salt bridge, are also satisfied by ApoE4 intermediate conformations in our simulations (S5 Table). Furthermore, R61 and E255 do not appear to have a direct role in inter-domain interaction as indicated by the inter-residue distance analysis. In this regard, an allosteric mechanism for ApoE4 inter-domain interaction [38] has been proposed as an alternative to the R61 and E255 salt bridge (Discussion).

## Discussion

Despite minimal differences in the primary structures (*i*.*e*., mutations of R158C in ApoE2, and C112R in ApoE4 with respect to most common ApoE3 isoform (Table 1)), the three ApoE variants show extremely divergent physiological (*i*.*e*., lipid binding [3,4,6,7]) and pathological (*i*.*e*., onset of AD [39,40]) behavior. Therefore, it is pivotal to understand how minimal mutations in the amino acid sequences determine such important and diverse functional differences among the three ApoE isoforms. Here, we investigate the folding mechanisms of all three ApoE variants in their monomeric form to identify specific structural determinants that could potentially be related to the physiological functions and pathological roles of each ApoE isoform.

### ApoE4 is less thermally stable than ApoE2 and ApoE3

In agreement with reported experimental melting temperatures [32,33], we observe that ApoE4 is less thermally stable than the other two isoforms (Fig 1). Although, in the current literature, it has been further recognized that ApoE2 is the most stable among the ApoE variants [32], we do not observe a clear differentiation between ApoE2 and ApoE3 thermal stability values in our study. However, such disparities between our *in silico* data and the reported experiments [32,33] can potentially be due to the presence of different ApoE oligomeric states in the experimental settings that could contribute differently to the overall stability of the protein. Further analysis of our simulations reveals that there are no significant differences in the loss of secondary structure with increasing temperature (S1B–S1G Fig), suggesting that the different mutations may potentially affect the tertiary structures of ApoE isoforms. Overall, we speculate that the differences in ApoE variants’ thermal stability can possibly be due to distinct populations of their respective conformational states.

### ApoE isoforms are characterized by different intermediate states

To identify representative structures of each ApoE isoform, we use PMF calculations and clustering analysis (Simulation analyses section in Methods). We observe an ApoE3 intermediate structure in which helix–1 and helix–4 together separate from helix–2 and helix–3 along with the opening of the N-terminal domain (Fig 3C). This structure is consistent with results reported by Fisher *et al*. showing that, in the N-terminal domain, helix–1 and helix–3 separate upon binding of lipids [41]. The identified structure is also consistent with the model proposed by Chen *et al*. for lipid association based on nuclear magnetic resonance data [16], as well as surface plasmon resonance data obtained by Nguyen *et al*. [7]. On the other hand, ApoE2 isoform is only characterized by an expansion of the N-terminal domain (Fig 3B), consistently with published data, which suggest the lack a well-defined intermediate state [5,33].

We also identify a unique ApoE4 misfolded intermediate state (Fig 3D), which may potentially play a pathological role in AD [18]. Compared to ApoE2 and ApoE3, the cluster in which we found the ApoE4 intermediate state exhibits the widest range in conformations (S10 Fig). We do not observe any increase in β-sheet content (S1E–S1G Fig) in the ApoE4 misfolded structure as reported by Morrow *et al*. [5]. However, we notice an increased value of radius of gyration (Fig 2I) in the identified ApoE4 misfolded intermediate state that is in stark agreement with previous studies reporting on the expanded volume of the helical N-terminal domain of this isoform [5,18]. Recently, Garai *et al*. have proposed a competitive binding mechanism between lipids and Aβ peptides to the C-terminal domain of each ApoE isoform. According to their hypothesis, ApoE monomers dissociate from the surface of the lipoprotein to bind Aβ assemblies [21]. Our simulations do not explicitly include lipids, yet, we speculate that the decreased flexibility of its C-terminal domain of ApoE4 misfolded intermediate state (S11 Fig) may facilitate the association of lipids and Aβ peptides. However, the elucidation of the structural mechanisms underlying these complex molecular events goes beyond the scope of the present study and will require further investigation.

### ApoE4 intermediate shows isoform-specific domain-domain interaction

Within the misfolded ApoE4 state, we observe a unique series of residue interactions (Fig 4I; Inter-domain interactions of ApoE isoforms in Results). Interestingly, ApoE4 exhibits the highest density of contacts between the N- and C-terminal domains in REX/DMD simulations, which is in agreement with the increased inter-domain interaction reported by Xu *et al*. as a unique feature of ApoE4 [42,43]. In our data, we recognize an alternative rearrangement of helix–1 (in the N-terminal domain) and C-terminal domain that is consistent with the FRET-based assay presented in the recent literature (S6 Fig) which report the activity of ApoE4 correctors in hindering the inter-domain interaction in ApoE4 [44,45]. Additionally, this interaction results in a trend where the ApoE4 intermediate conformation exhibits decreased hydrophobic solvent accessible surface area relative to ApoE2 and ApoE3 (S6 Table). We identify residues 196 to 215 in the hinge region and residues 235 to 243 in the C-terminal domain as important sites for domain-domain interactions. Our data overlap with observations from previous experiments consisting in multiple ApoE4 truncations and revealing that the region containing residues 166 through 259 is critical for inter-domain interaction [46]. Additionally, Zhang *et al*.’s findings confirm that the hinge region (residues 166 to 205) is specifically required for inter-domain interaction despite the two domains’ ability to fold independently [27,33]. In addition to physically joining the N- and C-terminal domains, the hinge region may also serve a direct role in inter-domain interaction as observed in our pair-wise distance analysis (see Results). In this context, we do not observe R61 and E255 as part of the residues involved in ApoE4 inter-domain interaction or in the salt bridge hypothesized to mediate this interaction (S9 Fig) [12,37]. However, the misfolded ApoE4 conformations observed in our simulations are in agreement with previously published experimental data (S5 Table). According to recent studies [38,47], the mutation C112R (*i*.*e*., from ApoE3 to ApoE4) generates a different distribution of charges along the N-terminal helix–4 in the latter isoform. Such change in ApoE4 may result in the rearrangement of the C-terminal domain with respect to the N-terminal helix bundle (*i*.*e*., inter-domain interaction) [47]. In this regard, Frieden *et al*. suggested that mutating R61 in ApoE4 to a non-charged (or oppositely charged) residue does not prevent the inter-domain interaction by breaking any salt-bridge with E255, but rather by reverting the charge distribution of ApoE4 N-terminal helix–4 to an ApoE3-like state [38]. Indeed, we observe a different distribution of charges, upon mutations of C112 and/or R61 in these two ApoE isoforms (S12 Fig). However, the elucidation of the allosteric mechanism underlying the inter-domain interaction goes beyond the scope of the present manuscript.

In conclusion, our simulations suggest that, the least thermally stable isoform ApoE4 may undergo the formation of an isoform-specific misfolded intermediate state with unique features such as inter-domain interactions (S7 Table). Based on recent literature [12,48–51], we speculate that this ApoE4 misfolded intermediate state may modify the lipid transport efficiency via an isoform-specific mechanism of interaction with lipids and lipoprotein receptors [1,3–7]. Concurrently, we surmise that the identified ApoE4-specific misfolded intermediate state might play a crucial role in the onset of AD by affecting the kinetic of aggregation or the clearance mechanisms of Aβ peptides [19–21] or by promoting the intracellular hyperphosphorylation and consequent self-assembly of tau protein [22,23,52–55]. Although, further studies will be required to confirm or exclude such possibilities (summarized in S13 Fig), we foresee the presented structural model of ApoE4 misfolded intermediate state as a new avenue to understanding AD pathogenesis, and to develop new pharmacological strategies [44,45] for probing the relationship between ApoE structure and function.

## Methods

### Simulation settings

Using our in house developed software Eris [34,35], we generate the starting structure of the ApoE3 isoform by re-introducing the five wild-type amino acids (*i*.*e*., Ala257Phe, Arg264Trp, Ala269Val, Gln279Leu, and Glu287Val) into the sequence of the recently published NMR structure of the monomeric ApoE3 mutant (PDB-ID: 2L7B [16]). The monomeric mutant ApoE3 from which we derive our starting structure shows nearly identical biophysical features (*i*.*e*., CD spectra[27], denaturation curves[27], DMPC clearance rate[27], competition for ^125^I-LDL binding to LDL receptor[27], and similar structures based on H/DX kinetics[56]) with respect to wild type variant. In the second stage, by introducing R158C and C112R mutations, we obtain the starting ApoE2 and ApoE4 structures, respectively. Additionally, using Eris, we evaluate the relative stability of the three ApoE isoforms by calculating their ΔΔG upon mutation. The results suggest that the mutation C112R (*i*.*e*., from ApoE3 to ApoE4) slightly destabilizes the protein structure with an estimated ΔΔG of 1.00 ± 0.52 kcal/mol. On the contrary, ApoE3 to ApoE2 is a neutral mutation with an estimated ΔΔG of -0.14 ± 0.41 kcal/mol.

At a later stage, we optimize the ApoE structures by means of short (*i*.*e*., 2x10^4^ DMD time steps, corresponding to ~1 ns) DMD simulations [28–30]. We evaluate DMD simulations’ ability to reproduce physiological phenomena by monitoring the occupancy of isoform-specific salt bridges. Several structural studies have reported that ApoE2 forms a salt bridge between R150 and D154, moreover ApoE4 contains a salt bridge between the two residues E109 and R112 [15,18,57]. Both of these isoform specific salt bridges are present in our simulations and support the ability of DMD to more accurately represent the ApoE isoforms (S9C Fig).

In DMD, atomic interactions (*i*.*e*., van der Waals and electrostatics) are approximated by multistep square-well potentials. We use a united atom representation for our all-atom protein models in which all heavy atoms and polar hydrogen atoms are explicitly represented. The simulation engine solves a series of two-body collisions, in which colliding atoms’ velocities change instantaneously according to the conservation laws of energy, momentum, and angular momentum. The Lazaridis-Karplus implicit solvation model [58] is adopted to account for the solvation energy. Temperature of the system is controlled with the Andersen thermostat [59]. We resolve any existing clashes in the protein structures using our in-house developed tool Chiron [60], and assess the quality of our lowest energy conformations using Gaia [61], our software that compares the intrinsic structural properties of *in silico* protein models to high-resolution crystal structures.

In our REX/DMD simulation, we use the replica exchange approach [62,63] in DMD simulations to efficiently explore the conformational landscape of the ApoE isoforms. In REX/DMD, multiple simulations of the same system at different temperatures (*i*.*e*., replicas) are performed in parallel. Replicas are periodically coupled through a Monte Carlo-based exchange of simulation temperatures allowing the system to easily overcome energetic barriers between minima in the free energy surface. For each isoform, we use 24 parallel replicas with temperatures ranging from 0.35 to 0.81 kcal/(mol k_B_) (corresponding to ~175 K and 405 K, respectively) with increments of 0.02 kcal/(mol k_B_)). We run the simulations for 6x10^6^ time steps (corresponding to approximately ~300 ns) per replica. Throughout the simulations, each replica visited an average of 13.9 +/- 4.8 of the different temperatures (S14 Fig). We determine when the simulations have reached equilibrium by monitoring the convergence of the ApoE isoform-specific heat capacity curves and concluded that our systems reach convergence near 4x10^6^ steps. We continued our simulations up to 6x10^6^ time steps to increase sampling and our ability to calculate accurate statistics (S15A–S15E Fig). Indeed, our simulations explore both native-like and misfolded “intermediate-like” N-terminal domain states (S16 Fig), while the C-terminal domain shows highly dynamic behavior, by exploring a very large ensemble of conformation around the N-terminal helix bundle (S17 Fig). The wall clock and CPU hours for each simulation are ~3,000 hours and ~71,000 hours respectively.

### Simulation analyses

We consider the first 5x10^5^ time steps of simulations as system equilibration, and omit them from our analyses. In order to determine the relative thermal stability of each ApoE isoform, we compute their heat capacities using the WHAM [64] for temperatures ranging from 0.4 to 0.8 kcal/(mol k_B_) (corresponding to ~200 K and ~400 K, respectively). WHAM analysis is performed through an *ad hoc* python script [65]. The retrieved heat capacity plots show local minima suggesting the presence of multiple intermediate states in the unfolding process of each ApoE isoform. We identify all of the ApoE isoform-specific states by calculating the PMF of each system under investigation. The validity of our PMF calculations is assessed by the normal distributions of potential energy at each REX/DMD temperature (S15F Fig). We choose the RMSD and Rg of the Cα atoms in the four helices constituting the ApoE N-terminal domain as reaction coordinates to identify protein conformational states using the following equation:

$$\text{A}\left(\text{RMSD},\text{Rg}\right)=-{\text{k}}_{\text{B}}\text{T ln}\left(\text{W}(\text{RMSD},\text{Rg})\right)-{\text{k}}_{\text{B}}\text{T ln}\left(\text{Z}\right)$$ (1)

where A is the Helmholtz free energy (kcal/mol), k_B_ is the Boltzmann constant (kcal/mol/K), T is the temperature (K), W is a function that defines the probability of a given pair of RMSD and Rg values and Z is the canonical partition function representing all possible conformational states of the protein. Since the second term of Eq 1 is a constant value, we derive the PMF as follows:

$$\text{PMF}\left(\text{RMSD},\text{Rg}\right)=-{\text{k}}_{\text{B}}\text{T ln}\left(\text{W}(\text{RMSD},\text{Rg})\right)+\text{C}$$ (2)

where the constant C sets the lowest PMF value at any given temperature to be zero. In our REX/DMD simulations, the C-terminal domain of all ApoE isoforms is highly flexible (S3 and S17 Figs). Therefore, we exclude it from the definition of our reaction coordinates to reduce the degeneracy of protein conformational states in the PMF calculations. RMSD, Rg and PMF were computed using GROMACS analysis tools [66].

Next, we isolate the most populated clusters of ApoE isoforms’ conformations from local minima on the PMF-derived free energy landscapes, and define their centroids as representative protein structures. We use the leader algorithm as implemented in Wordom [67,68] for clustering analysis, using a cutoff defined by the highest peak value in the distribution of pairwise RMSDs of the Cα atoms in the four helices of the ApoE N-terminal domain.

Additionally, to characterize the structural features of each ApoE isoform, we monitor the secondary structure content in our REX/DMD simulations at different temperatures using Wordom (S1B–S1G Fig) [67,68]. Furthermore, we investigate the inter-domain interactions in each ApoE isoform using *ad hoc* scripts to compute matrices of pairwise distances between Cα atoms.

## Supporting Information

S1 Fig Secondary structure analyses of ApoE isoforms. (A) Cartoon representation of ApoE. The same structural features are applicable for all three ApoE isoforms: helix–1 (H1, residues 24 to 41), helix–2 (H2, residues 55 to 80), helix–3 (H3, residues 90 to 125), helix–4 (H4, residues 131 to 165), hinge region (residues 166 to 205) and C-terminal domain (residues 206 to 299) are represented in purple, green, blue, red, and grey, respectively. Single residue secondary structure analysis of ApoE2 (B), ApoE3 (C), and ApoE4 (D). The probability of secondary structure content (indicated in the plot as H, B, and L for alpha-helix, beta strand, and disordered respectively) at a specific residue is proportional to the relative height of the letter at that site. The same color code of (A) is used to indicate the helices position in (B-D). The average percentage of secondary structure content (alpha helix, beta strand, and disordered) as a function of temperatures T1 (~275 K for all three ApoE isoforms), T2 (~321 K, ~318 K, and ~309 K for ApoE2 (E), ApoE3 (F) and ApoE4 (G) respectively), T3 (~340 K, ~338 K, and ~328 K for ApoE2, ApoE3 and ApoE4 respectively) and T4 (~355 K, ~365 K, and ~342 K for ApoE2, ApoE3, and ApoE4, respectively) reveal that all of the three isoforms lose secondary structure without significant differences.
(TIF)

S2 Fig Representative structures of ApoE isoforms. Representative structures (i.e., centroid of the most populated cluster) from clustering analysis of the ApoE isoforms’ conformations extracted from the free energy basin (A-C) at T1 (~275 K for all three ApoE isoforms) and (D-F) at T2 (~321 K, ~318 K, and ~309 K for ApoE2, ApoE3 and ApoE4 respectively). All three isoforms exhibit native-live conformations with compact N-terminal domains. (G-I) at T3 (~340 K, ~338 K, and ~328 K for ApoE2, ApoE3 and ApoE4 respectively), the intermediate states of each ApoE variant represent the dominant conformations in the free energy landscape (see Fig 2 in the main text). (J-L) at T4 (~355 K, ~365 K, and ~342 K for ApoE2, ApoE3, and ApoE4, respectively), the tertiary contacts are lost with the complete unfolding of the proteins. The size of the most populated cluster is reported in each panel. For all structures, helix–1 (H1), helix–2 (H2), helix–3 (H3), and helix–4 (H4) are represented in purple, green, blue, and red, cartoon respectively. The rest of the protein is represented in grey cartoon.
(TIF)

S3 Fig RMSD distributions of ApoE isoforms’ domains. Distributions of the RMSD of the Cα atoms for ApoE2 (A), ApoE3 (B), and ApoE4 (C) isoform at T1 (~275 K for all three ApoE isoforms), T2 (~321 K, ~318 K, and ~309 K for ApoE2, ApoE3 and ApoE4 respectively), T3 (~340 K, ~338 K, and ~328 K for ApoE2, ApoE3 and ApoE4 respectively) and T4 (~355 K, ~365 K, and ~342 K for ApoE2, ApoE3, and ApoE4, respectively) for the full protein (residues 1 to 299 in black), the N-terminal domain (residues 1 to 165 in red), and the C-terminal domain including the hinge region (residues 166 to 299 in blue). For temperatures ranging from T1 to T3, the C-terminal domain exhibits larger RMSD values than the N-terminal domain. At temperature T4 the RMSD values for N-terminal domain are much larger and comparable with the C-terminal domain indicating the overall unfolding of the protein. Temperatures from T1 to T4 are reported as insets within each plot. For all histograms, the width of the bins corresponds to 1 Å.
(TIF)

S4 Fig N-terminal comparison of REX/DMD centroids with crystal structures. Each panel represents an alignment between the centroids isolated through clustering analysis at 275 K from REX/DMD simulations with different previously solved crystal structures. (A) ApoE2 (1LE2) (B) ApoE3 (1NFN) (C) ApoE3 (1OR3) (D) ApoE3 (1OR2) (E) ApoE3 (1LPE) (F) ApoE3 (1BZ4) (G) ApoE4 (1B68) (H) ApoE4 (1GS9) (I) ApoE4 (1LE4). The corresponding RMSD for each alignment is presented in S3 Table. Structures in grey cartoon are crystallographic and structures in blue cartoon are the N-terminal domains as shown in S2A–S2C Fig.
(TIF)

S5 Fig ApoE native conformation contact maps. Contact maps were calculated for the ApoE native-like conformations of each isoform isolated from clustering analysis at 275 K. A comparison was made with X-ray crystal structures of ApoE isoforms seen in S2 Table and S4 Fig. The distances were measured in angstroms between Cα atoms of residues 24–82 and residues 93–162, the residues common amongst all structures. The numbers along the x- and y-axes represent the residue numbers. The color bar represents the distance between the centroid computed over the residues’ side chains in Å.
(TIF)

S6 Fig End to end distances in ApoE4 misfolded intermediate state. The histogram illustrates the distribution of distances measured between the Cα atoms in terminal residues K1 and H299 in ApoE4 from serial DMD simulations. Two single temperature DMD simulations were performed at 309 K for 1 million time steps (~50 ns) using our ApoE4 misfolded intermediate state as a starting structure. The two different starting structures correspond to the centroid of the most populated cluster (S2I Fig) and the lowest energy structure from the same cluster. The histogram reveals that the two termini in the ApoE4 misfolded intermediate conformation are below 60 Å for the majority of the simulations. This is a rough estimate for potential to generate FRET signals. The width of the histogram bins corresponds to 1 Å.
(TIF)

S7 Fig Pair-wise inter-residue distances in the ApoE4 misfolded intermediate state. Inter-residue distance analysis of the most populated cluster of ApoE4 conformations extracted from the free energy basin at T3 (~342 K, see Fig 2I) reveals a unique series of contacts (outline in red) and reported in S8A Fig. The upper and lower triangular matrices represent respectively the average and the standard deviation of the pair-wise inter-residue distance in Å. The color bar represents the distance between the centroid computed over the residues’ side chains in Å.
(TIF)

S8 Fig Domain-domain interaction as a unique feature of the ApoE4 misfolded intermediate state. (A) Inter-residue contacts (residues from 26 to 44, from 196 to 215, and from 235 to 243) identified in the ApoE4 misfolded intermediate state at temperature T3 (~342 K). The relative positions of the same residues is reported for ApoE2 (B) and ApoE3 (C) at temperature T3 (~340 K and ~338 K, respectively). For all structures, helix–1 (H1), helix–2 (H2), helix–3 (H3), and helix–4 (H4) are represented in purple, green, blue, and red, cartoon respectively. Residues in contacts are reported as yellow cartoon (side chains represented as sticks only for ApoE4), while the rest of the protein is represented in grey cartoon.
(TIF)

S9 Fig Putative salt bridge distance in ApoE4. (A) The distribution of distances between residues R61 and E255 as explored in ApoE4 isoform REX/DMD simulations is consistently greater than 20 Å regardless of the simulation temperature, and thus, not compatible with the existence of a salt bridge between the two residues. The width of the histogram bins corresponds to 1 Å. (B) A cartoon representation of the putative salt bridge interaction adapted from Mahley RW, Huang Y (2012) J Med Chem, 55: 8997–9008. doi:10.1021/jm3008618. (C) Occupancy rate of isoform-specific salt bridges.
(TIF)

S10 Fig RMSD distributions of ApoE Intermediate state clusters. For the most populated cluster of each ApoE isoform at T3 (~340 K, ~338 K, and ~328 K for ApoE2, ApoE3 and ApoE4, respectively), the RMSD between each member of the cluster and the centroid was calculated. The width of histogram bins corresponds to 1 Å RMSD of the Cα atoms in the N-terminal domain helices.
(TIF)

S11 Fig C-terminal flexibility within ApoE intermediate states. Distances between the center of mass of three different segments of the C-terminal domains (i.e, residues 206 to 216, residues 247 to 257, and residues 289 to 299) and the center of mass of the helix–4 in the N-terminal domain for each ApoE isoforms. Being the most stable helix in all ApoE simulations (see S1B–S1D Fig), helix–4 has been chosen as reference position in the N-terminal domain. All calculations performed on the most populated clusters at temperature T3 (~340 K, ~338 K, and ~328 K for ApoE2, ApoE3 and ApoE4, respectively). For all histograms, the width of the bins corresponds to 1 Å.
(TIF)

S12 Fig Electrostatic surface potential in ApoE3 and ApoE4 isoforms. Comparisons of the electrostatic surface potential of the N-terminal domain of ApoE3, ApoE4 and the ApoE4-R61T. Blue and red colors correspond to positively and negatively charged surfaces, respectively, and white color corresponds the neutral hydrophobic ones.
(TIF)

S13 Fig Potential ApoE4-related pathological pathways. We speculate that the ApoE4 misfolded intermediate state may affect the kinetics of Aβ peptides aggregation and clearance. Concurrently, it may be favor the formation of intracellular neurofibrillary tangles. Further studies are required to elucidate the molecular mechanisms underlying the intracellular events leading to tau hyperphosphorylation and aggregation. Moreover, additional investigations are necessary to elucidate the molecular events at the basis of the interaction between ApoE4, lipids, and Aβ peptides.
(TIF)

S14 Fig REX/DMD Replicas in temperature space. Representative replicas from our ApoE3 REX/DMD simulations are plots with their DMD temperature (kcal/mol/k_B_) as a function of time steps. There are replicas that travel through a wide range of temperature and those that are confined to a smaller spread in temperature space.
(TIF)

S15 Fig Heat capacity convergence and energy distributions in REX/DMD simulations of ApoE isoforms. The heat capacity (Cv) curves for ApoE2 (A), ApoE3 (B), and ApoE4 (C) isoforms computed using WHAM on REX/DMD trajectories in the range of 200 to 400 K including 5.5x10^6^, 4.5x10^6^, 3.5x10^6^ and 2.5x10^6^ time steps show the convergence of REX/DMD simulations. The heat capacity curves computed using WHAM on REX/DMD trajectories for ApoE4 in the range of 275K to 400K (D) using two independent and equal size windows from the same simulation. The peak positions in the two curves are slightly shifted revealing that 3x10^6^ time steps is not sufficient to reach convergence. (E) The Cv curves computed using different windows corresponding to 4x10^6^ time steps from the ApoE4 REX/DMD simulation. Segments of 4x10^6^ time steps in WHAM calculations allow for more consistent peak locations. (F) The three ApoE isoforms exhibit Gaussian distributions of potential energy supporting the treatment of REX/DMD simulations as partition functions at T1 (~275 K for all three ApoE isoforms), T2 (~321 K, ~318 K, and ~309 K for ApoE2, ApoE3 and ApoE4 respectively), T3 (~340 K, ~338 K, and ~328 K for ApoE2, ApoE3 and ApoE4 respectively) and T4 (~355 K, ~365 K, and ~342 K for ApoE2, ApoE3, and ApoE4, respectively). The width of the histogram bins corresponds to 1 kcal/mol.
(TIF)

S16 Fig “Native-like” and “intermediate-like” N-terminal states of ApoE4. (A) The RMSD of the Cα atoms in the N-terminal domain helices was calculated between the trajectory of conformations at 322 K and the centroids found from clustering analysis at 309 K (T2) for the “native-like” state and at 328 K (T3) for the misfolded “intermediate-like” state. The RMSD values reveal that at the transition peak, ApoE4 visits both the “native-like” and misfolded “intermediate-like” N-terminal domain conformations (RMSD < 5 Å). (B) The conformations found at the transition state peak with the lowest RMSD values from (A) shown in blue are aligned with their corresponding centroid structures colored in gray. The “native-like” conformation has an RMSD value of 3.28 Å with its respective centroid while the “intermediate-like” conformation has an RMSD value of 5.68 Å suggesting that the same conformation is found in the free energy basin at 328 K. The RMSD for both (A) and (B) is calculated using the N-terminal domain helices because the flexibility of the C-terminal domain adds considerable noise as discussed in the Methods section and as shown in S3 Fig.
(TIF)

S17 Fig C-terminal conformations in ApoE4. (A) The superposition of ApoE4 C-terminal conformations from REX/DMD simulations at 309 K with an aligned N-terminal domain reveals that the C-terminal domain explores a variety of positions relative to the N-terminal domain. The C-terminal domain assumes conformations next to the helix–1/helix–2 side of the N-terminal domain as suggested previously in literature as well as in conformations next to the helix–1/helix–4 and helix–4/helix–3 sides. The superposition of every 25^th^ frame of C-terminal conformations from the REX/DMD trajectory is represented in grey. A representative conformation of the N-terminal domain alignment is shown with helix–1 (H1), helix–2 (H2), helix–3 (H3), and helix–4 (H4) in purple, green, blue, and red, cartoon, respectively. (B) The angle between the N-terminal domain and C-terminal domain shows the relative closeness between the two domains. An angle of zero degrees represents the N-terminal and C-terminal next to each other, while an angle of 180 degrees represents conformations with the C-terminal away from the sides of the N-terminal domain. The angle *θ* is measured using residues L148 and G165 in the most stable N-terminal helix, helix–4, and L252 representing the center of the most stable C-terminal domain helix. Note that the magnitude of the angle does not always correspond to a similar magnitude in distances between the two domains. (C) The dihedral angle φ between the N-terminal domain and the C-terminal domain shows the relative orientation between the two domains. An angle of zero degrees represents an anti-parallel orientation between the two domain helices while an angle of -180 or 180 degrees represents a parallel orientation. N-terminus to C-terminus is used for directionality. The angle φ is measured between vectors defined by the center of mass of residues E131 and G165 in the most stable N-terminal helix, helix–4, and residues E238 and F265 in the most stable C-terminal domain helix. The width of the histogram bins corresponds to 1 degree.
(TIF)

S1 Table Human ApoE sequence. (DOCX)

S2 Table Structures found in our simulations have N-terminal helix conformations similar to solved crystal structures. (DOCX)

S3 Table RMSD between native-like ApoE conformations at 275 K. (DOCX)

S4 Table Population of ApoE intermediates at physiological temperatures. (DOCX)

S5 Table EPR distances in REX/DMD simulations. (DOCX)

S6 Table Hydrophobic solvent exposed surface area of ApoE intermediate states. (DOCX)

S7 Table ApoE structural and thermodynamic insights. (DOCX)

S1 Text Supporting references. (DOCX)
