# Supplementary figures and images for: ApoE4-specific Misfolded Intermediate Identified by Molecular Dynamics Simulations
Source: PLoS Comput Biol. 2015 Oct 27;11(10):e1004359. doi: 10.1371/journal.pcbi.1004359 (PMC4623519; doi:10.1371/journal.pcbi.1004359)

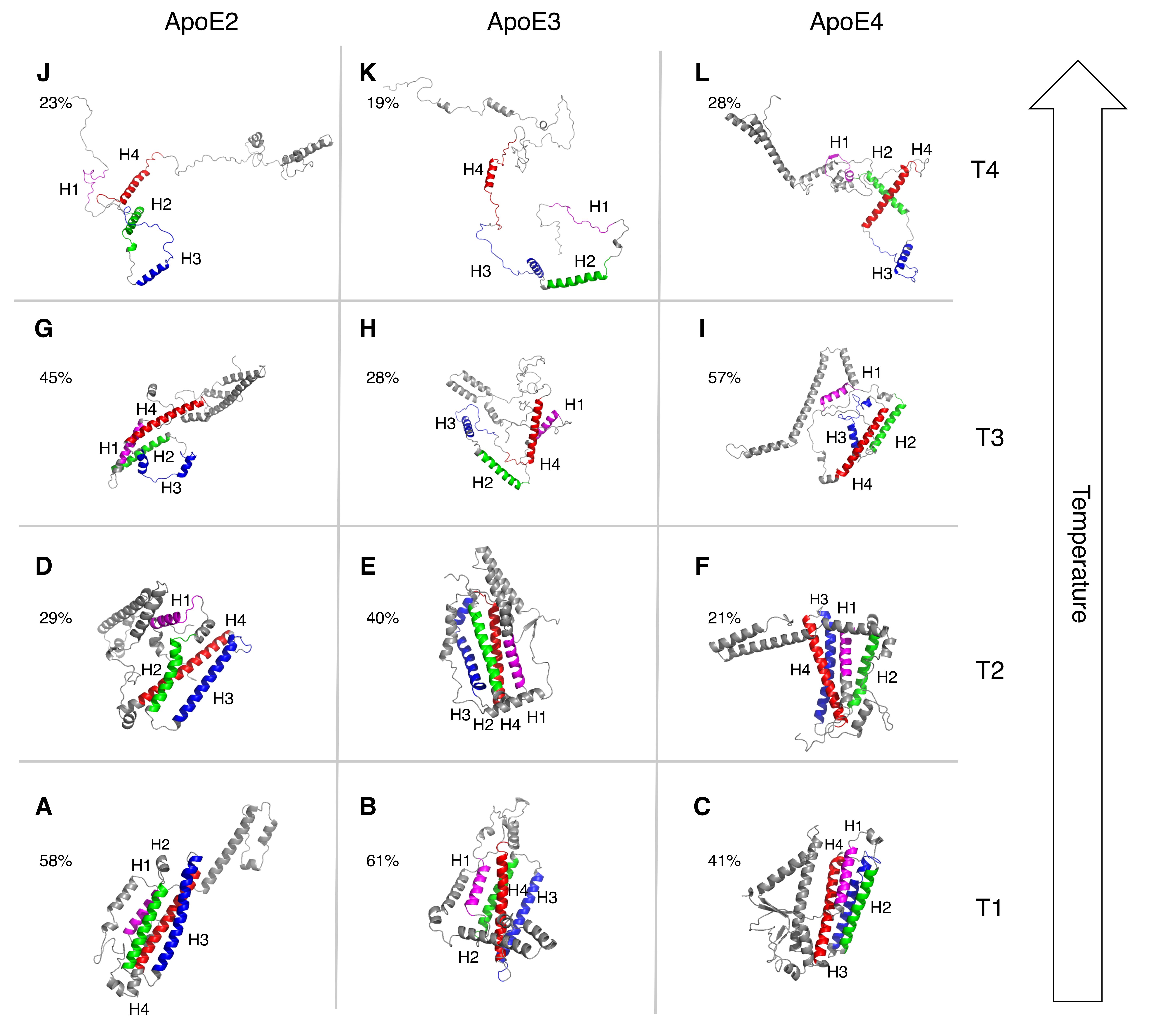

Supplement: S2 Fig — Representative structures (i.e., centroid of the most populated cluster) from clustering analysis of the ApoE isoforms’ conformations extracted from the free energy basin (A-C) at T1 (~275 K for all three ApoE isoforms) and (D-F) at T2 (~321 K, ~318 K, and ~309 K for ApoE2, ApoE3 and ApoE4 respectively). All three isoforms exhibit native-live conformations with compact N-terminal domains. (G-I) at T3 (~340 K, ~338 K, and ~328 K for ApoE2, ApoE3 and ApoE4 respectively), the intermediate states of each ApoE variant represent the dominant conformations in the free energy landscape (see Fig 2 in the main text). (J-L) at T4 (~355 K, ~365 K, and ~342 K for ApoE2, ApoE3, and ApoE4, respectively), the tertiary contacts are lost with the complete unfolding of the proteins. The size of the most populated cluster is reported in each panel. For all structures, helix–1 (H1), helix–2 (H2), helix–3 (H3), and helix–4 (H4) are represented in purple, green, blue, and red, cartoon respectively. The rest of the protein is represented in grey cartoon. (TIF) [file pcbi.1004359.s002.tif]

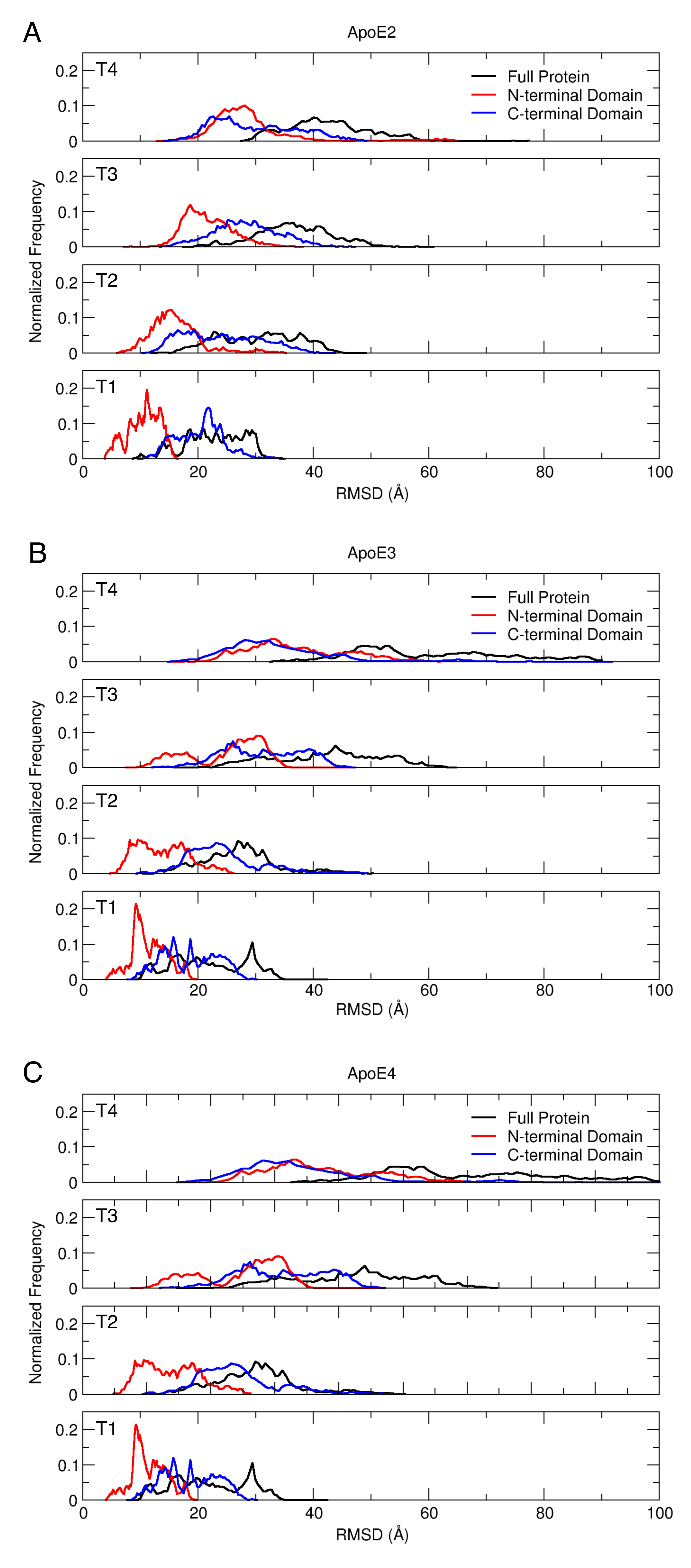

Supplement: S3 Fig — Distributions of the RMSD of the Cα atoms for ApoE2 (A), ApoE3 (B), and ApoE4 (C) isoform at T1 (~275 K for all three ApoE isoforms), T2 (~321 K, ~318 K, and ~309 K for ApoE2, ApoE3 and ApoE4 respectively), T3 (~340 K, ~338 K, and ~328 K for ApoE2, ApoE3 and ApoE4 respectively) and T4 (~355 K, ~365 K, and ~342 K for ApoE2, ApoE3, and ApoE4, respectively) for the full protein (residues 1 to 299 in black), the N-terminal domain (residues 1 to 165 in red), and the C-terminal domain including the hinge region (residues 166 to 299 in blue). For temperatures ranging from T1 to T3, the C-terminal domain exhibits larger RMSD values than the N-terminal domain. At temperature T4 the RMSD values for N-terminal domain are much larger and comparable with the C-terminal domain indicating the overall unfolding of the protein. Temperatures from T1 to T4 are reported as insets within each plot. For all histograms, the width of the bins corresponds to 1 Å. (TIF) [file pcbi.1004359.s003.tif]

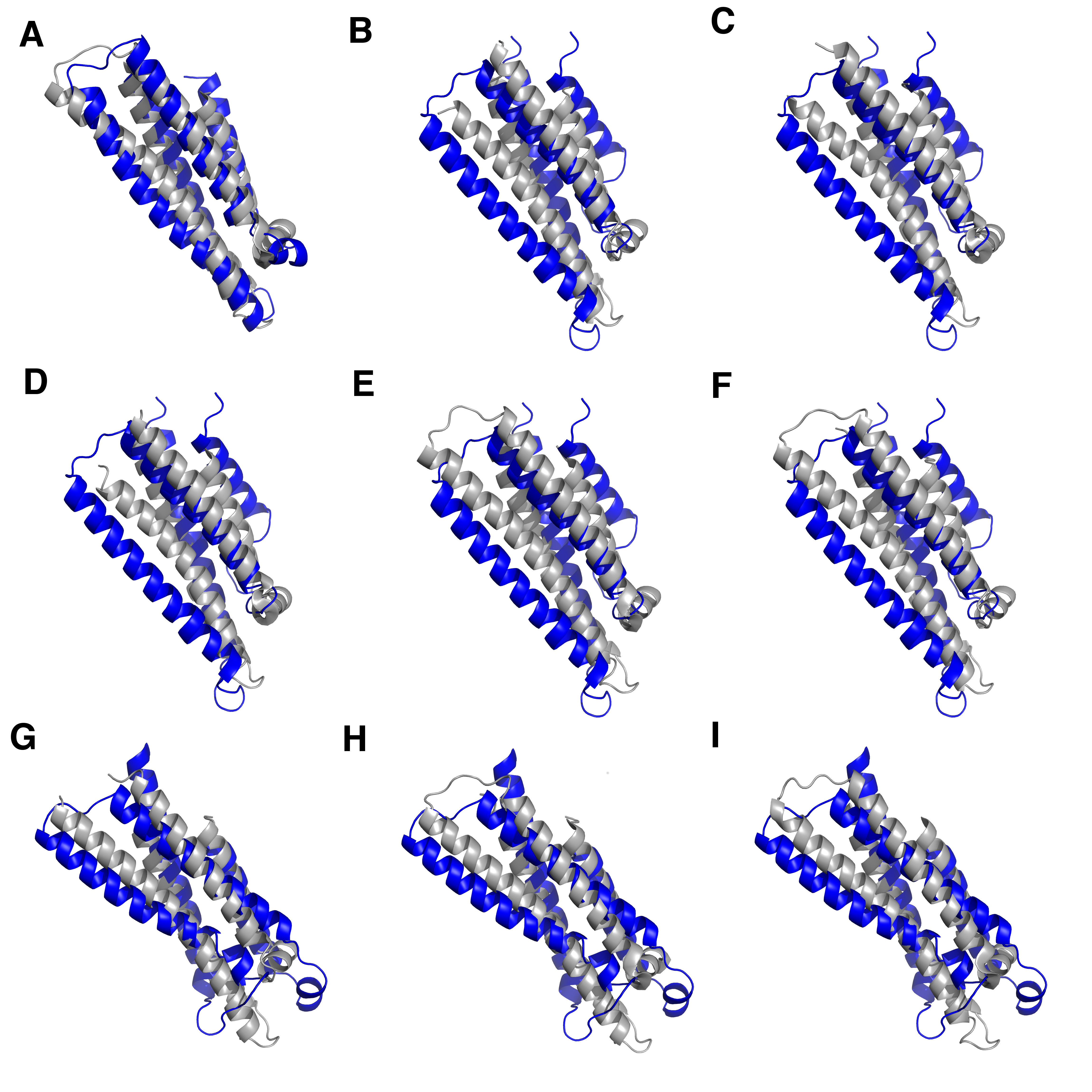

Supplement: S4 Fig — Each panel represents an alignment between the centroids isolated through clustering analysis at 275 K from REX/DMD simulations with different previously solved crystal structures. (A) ApoE2 (1LE2) (B) ApoE3 (1NFN) (C) ApoE3 (1OR3) (D) ApoE3 (1OR2) (E) ApoE3 (1LPE) (F) ApoE3 (1BZ4) (G) ApoE4 (1B68) (H) ApoE4 (1GS9) (I) ApoE4 (1LE4). The corresponding RMSD for each alignment is presented in S3 Table. Structures in grey cartoon are crystallographic and structures in blue cartoon are the N-terminal domains as shown in S2A–S2C Fig. (TIF) [file pcbi.1004359.s004.tif]

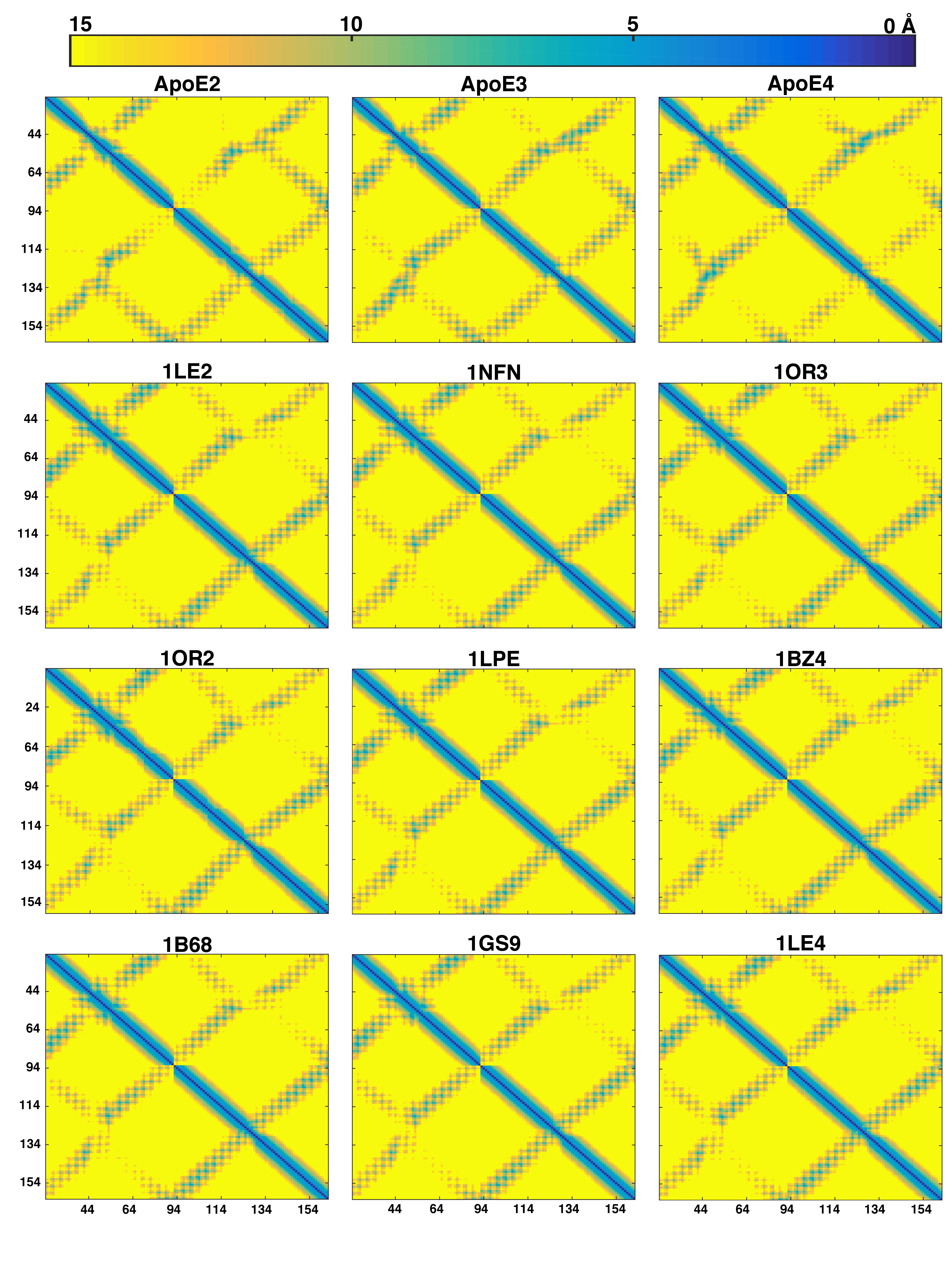

Supplement: S5 Fig — Contact maps were calculated for the ApoE native-like conformations of each isoform isolated from clustering analysis at 275 K. A comparison was made with X-ray crystal structures of ApoE isoforms seen in S2 Table and S4 Fig. The distances were measured in angstroms between Cα atoms of residues 24–82 and residues 93–162, the residues common amongst all structures. The numbers along the x- and y-axes represent the residue numbers. The color bar represents the distance between the centroid computed over the residues’ side chains in Å. (TIF) [file pcbi.1004359.s005.tif]

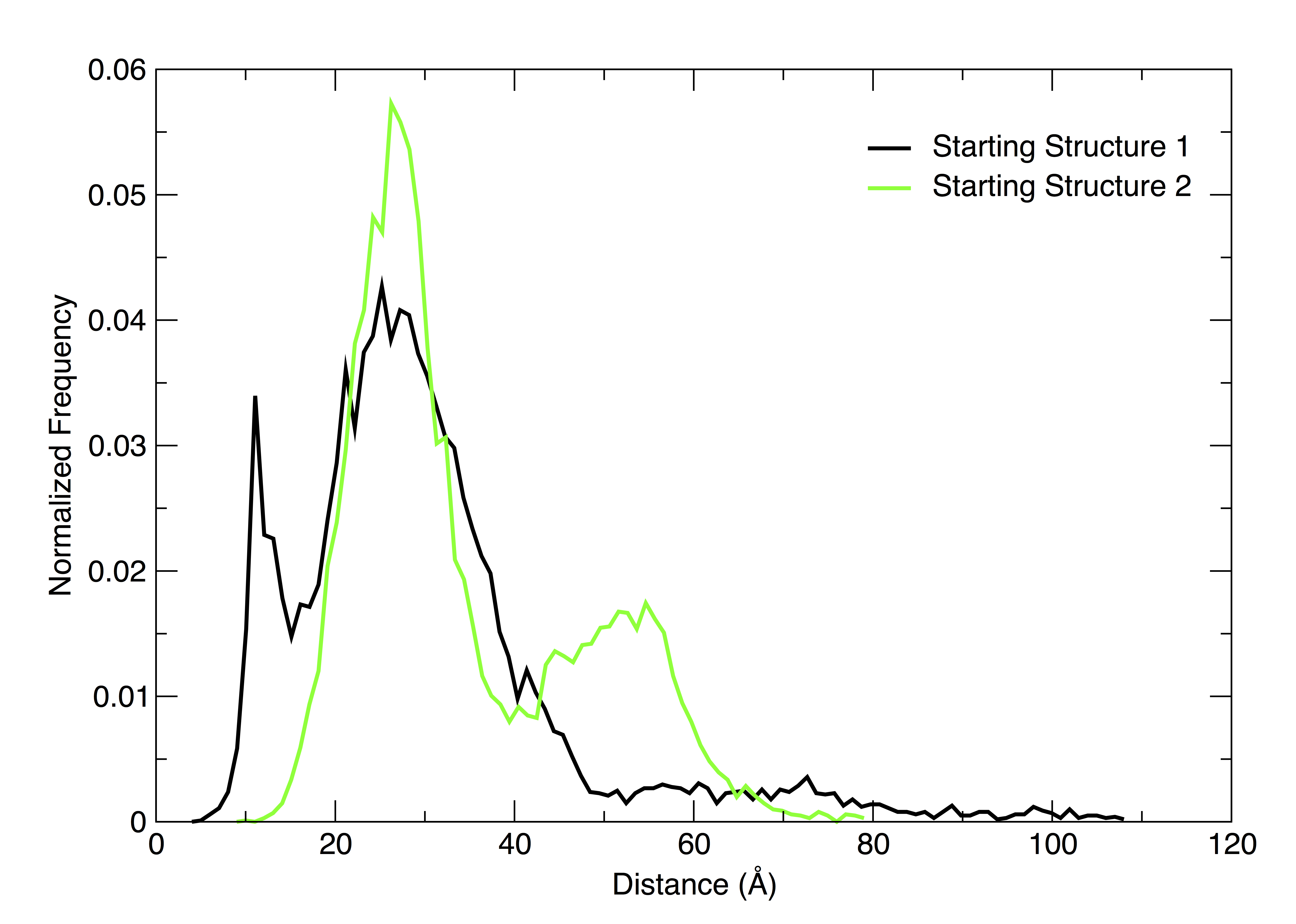

Supplement: S6 Fig — The histogram illustrates the distribution of distances measured between the Cα atoms in terminal residues K1 and H299 in ApoE4 from serial DMD simulations. Two single temperature DMD simulations were performed at 309 K for 1 million time steps (~50 ns) using our ApoE4 misfolded intermediate state as a starting structure. The two different starting structures correspond to the centroid of the most populated cluster (S2I Fig) and the lowest energy structure from the same cluster. The histogram reveals that the two termini in the ApoE4 misfolded intermediate conformation are below 60 Å for the majority of the simulations. This is a rough estimate for potential to generate FRET signals. The width of the histogram bins corresponds to 1 Å. (TIF) [file pcbi.1004359.s006.tif]

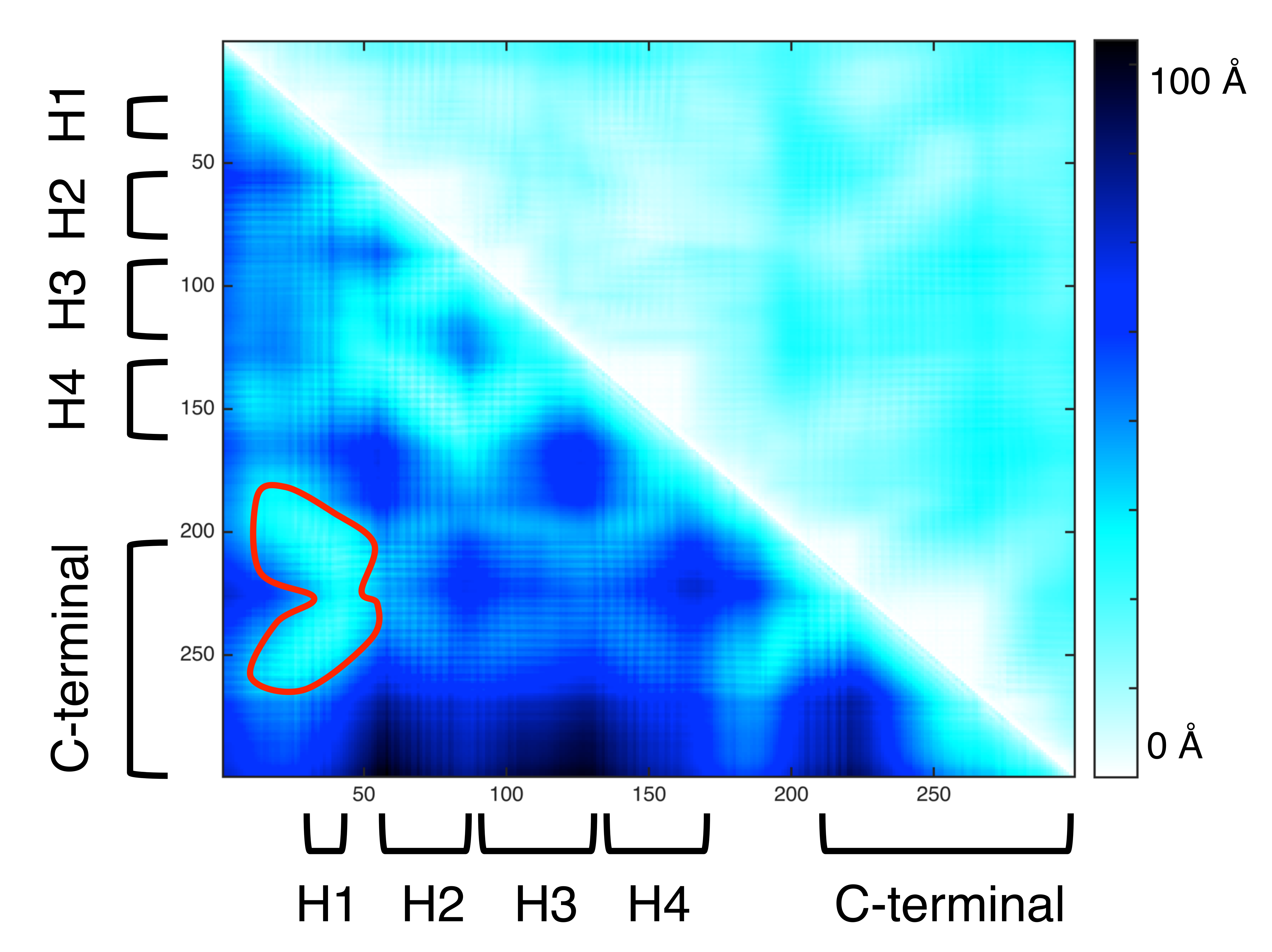

Supplement: S7 Fig — Inter-residue distance analysis of the most populated cluster of ApoE4 conformations extracted from the free energy basin at T3 (~342 K, see Fig 2I) reveals a unique series of contacts (outline in red) and reported in S8A Fig. The upper and lower triangular matrices represent respectively the average and the standard deviation of the pair-wise inter-residue distance in Å. The color bar represents the distance between the centroid computed over the residues’ side chains in Å. (TIF) [file pcbi.1004359.s007.tif]

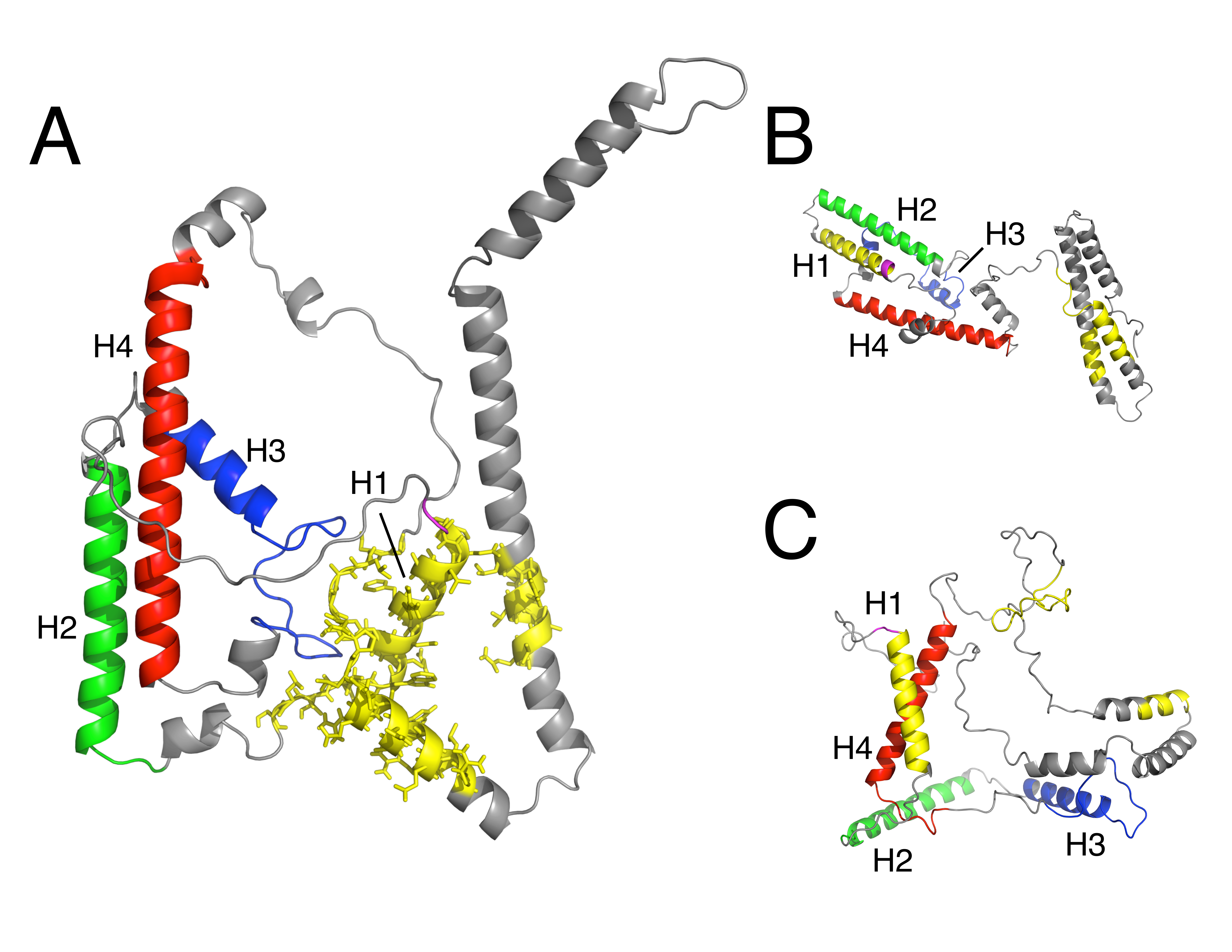

Supplement: S8 Fig — (A) Inter-residue contacts (residues from 26 to 44, from 196 to 215, and from 235 to 243) identified in the ApoE4 misfolded intermediate state at temperature T3 (~342 K). The relative positions of the same residues is reported for ApoE2 (B) and ApoE3 (C) at temperature T3 (~340 K and ~338 K, respectively). For all structures, helix–1 (H1), helix–2 (H2), helix–3 (H3), and helix–4 (H4) are represented in purple, green, blue, and red, cartoon respectively. Residues in contacts are reported as yellow cartoon (side chains represented as sticks only for ApoE4), while the rest of the protein is represented in grey cartoon. (TIF) [file pcbi.1004359.s008.tif]

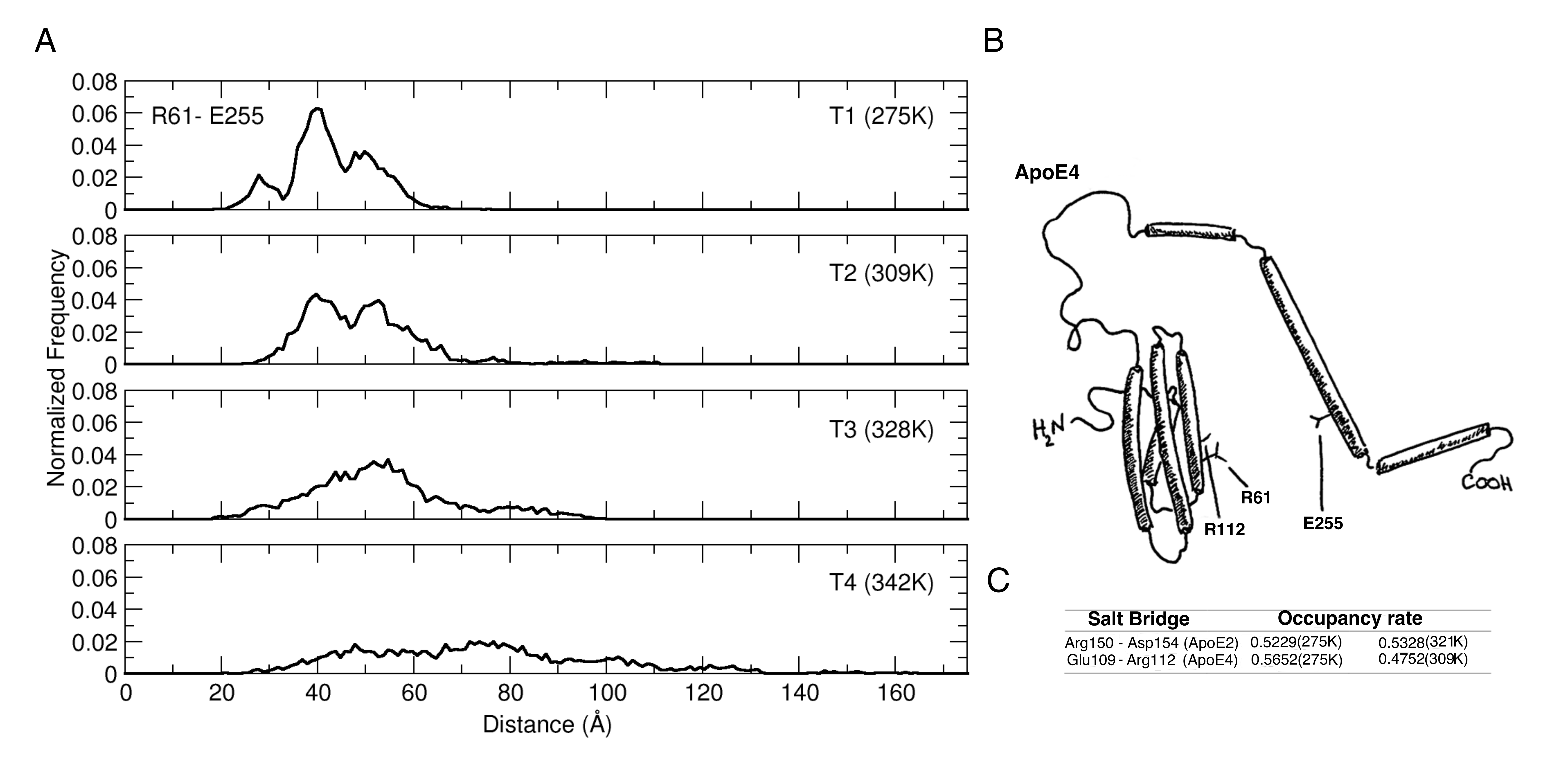

Supplement: S9 Fig — (A) The distribution of distances between residues R61 and E255 as explored in ApoE4 isoform REX/DMD simulations is consistently greater than 20 Å regardless of the simulation temperature, and thus, not compatible with the existence of a salt bridge between the two residues. The width of the histogram bins corresponds to 1 Å. (B) A cartoon representation of the putative salt bridge interaction adapted from Mahley RW, Huang Y (2012) J Med Chem, 55: 8997–9008. doi:10.1021/jm3008618. (C) Occupancy rate of isoform-specific salt bridges. (TIF) [file pcbi.1004359.s009.tif]

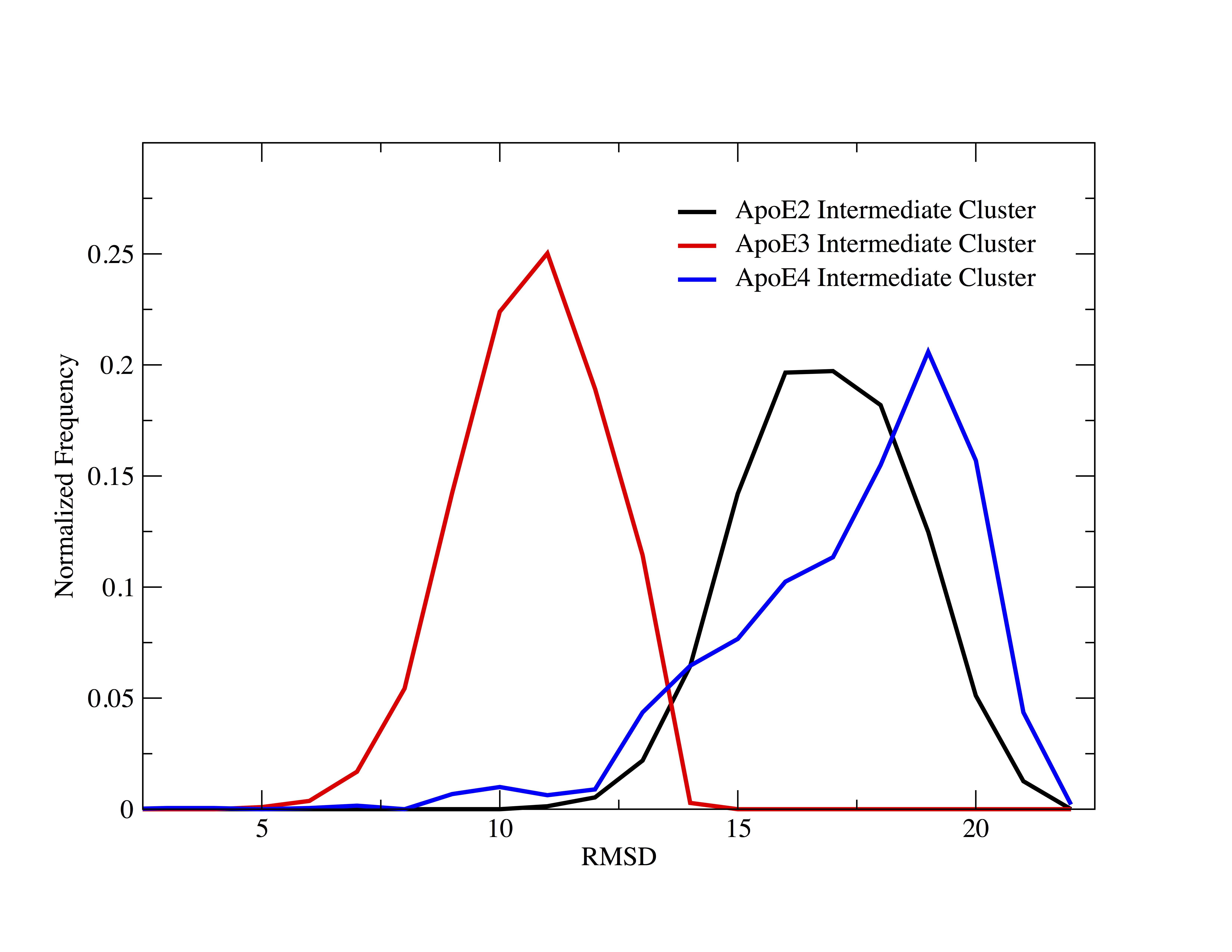

Supplement: S10 Fig — For the most populated cluster of each ApoE isoform at T3 (~340 K, ~338 K, and ~328 K for ApoE2, ApoE3 and ApoE4, respectively), the RMSD between each member of the cluster and the centroid was calculated. The width of histogram bins corresponds to 1 Å RMSD of the Cα atoms in the N-terminal domain helices. (TIF) [file pcbi.1004359.s010.tif]

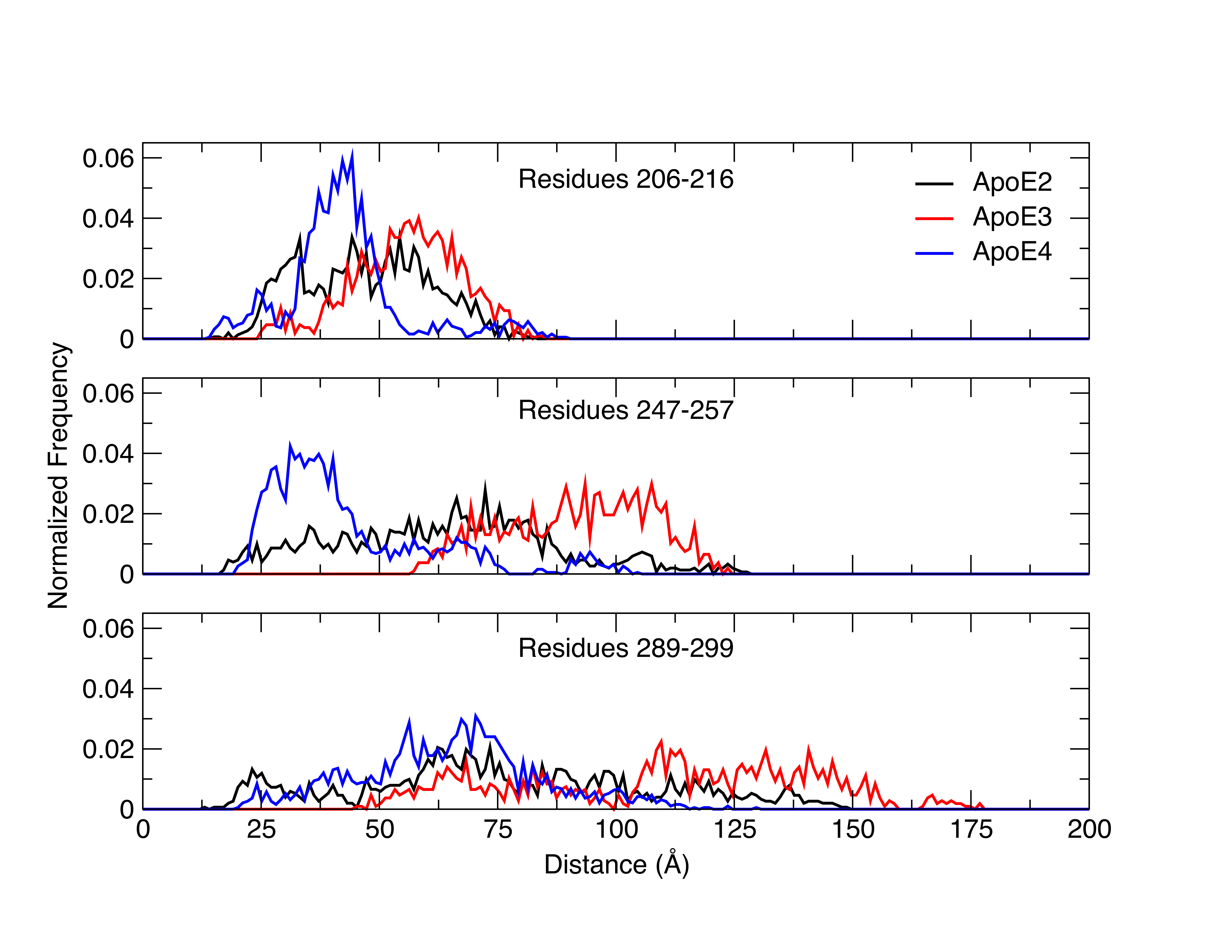

Supplement: S11 Fig — Distances between the center of mass of three different segments of the C-terminal domains (i.e, residues 206 to 216, residues 247 to 257, and residues 289 to 299) and the center of mass of the helix–4 in the N-terminal domain for each ApoE isoforms. Being the most stable helix in all ApoE simulations (see S1B–S1D Fig), helix–4 has been chosen as reference position in the N-terminal domain. All calculations performed on the most populated clusters at temperature T3 (~340 K, ~338 K, and ~328 K for ApoE2, ApoE3 and ApoE4, respectively). For all histograms, the width of the bins corresponds to 1 Å. (TIF) [file pcbi.1004359.s011.tif]

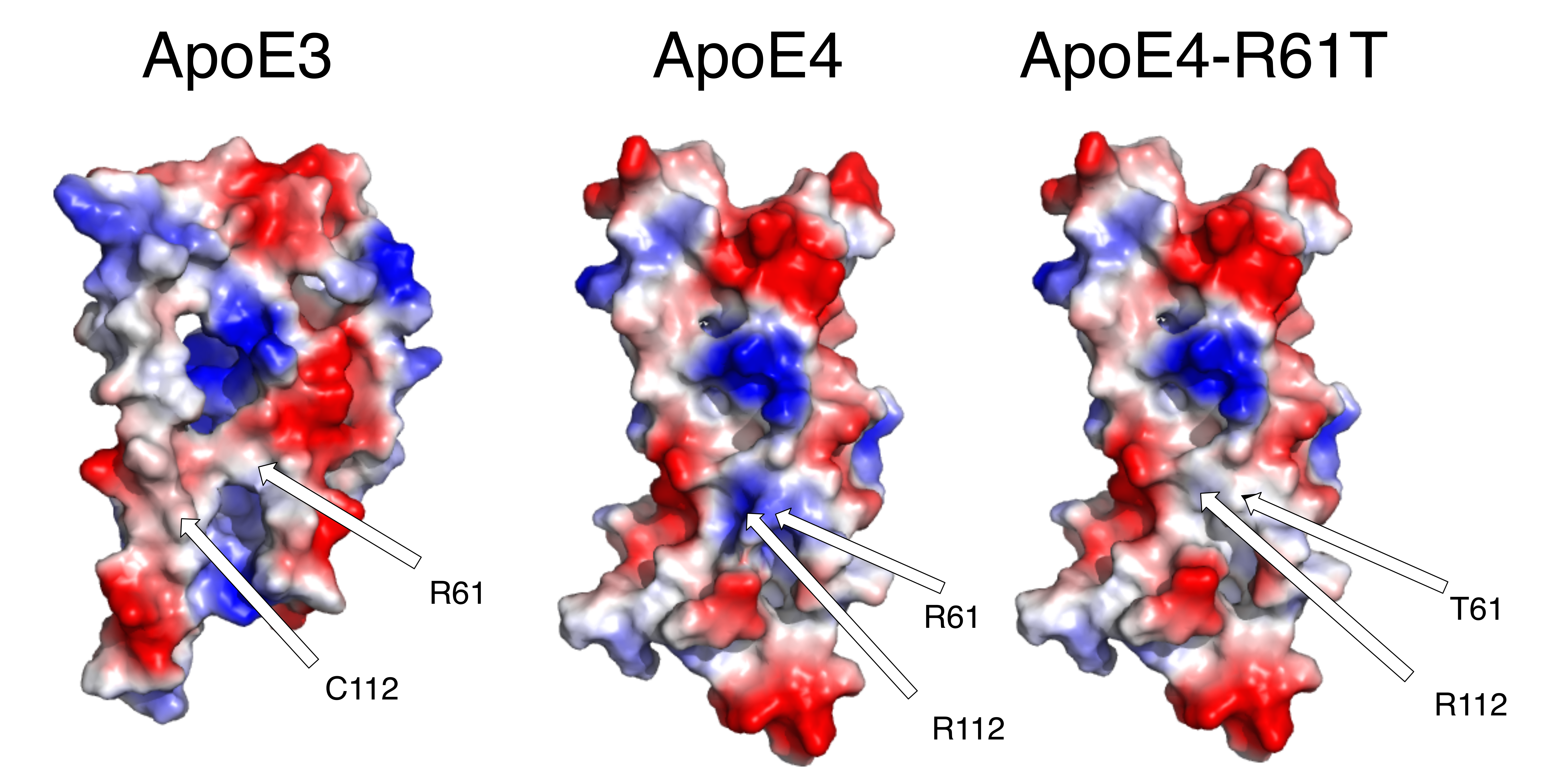

Supplement: S12 Fig — Comparisons of the electrostatic surface potential of the N-terminal domain of ApoE3, ApoE4 and the ApoE4-R61T. Blue and red colors correspond to positively and negatively charged surfaces, respectively, and white color corresponds the neutral hydrophobic ones. (TIF) [file pcbi.1004359.s012.tif]

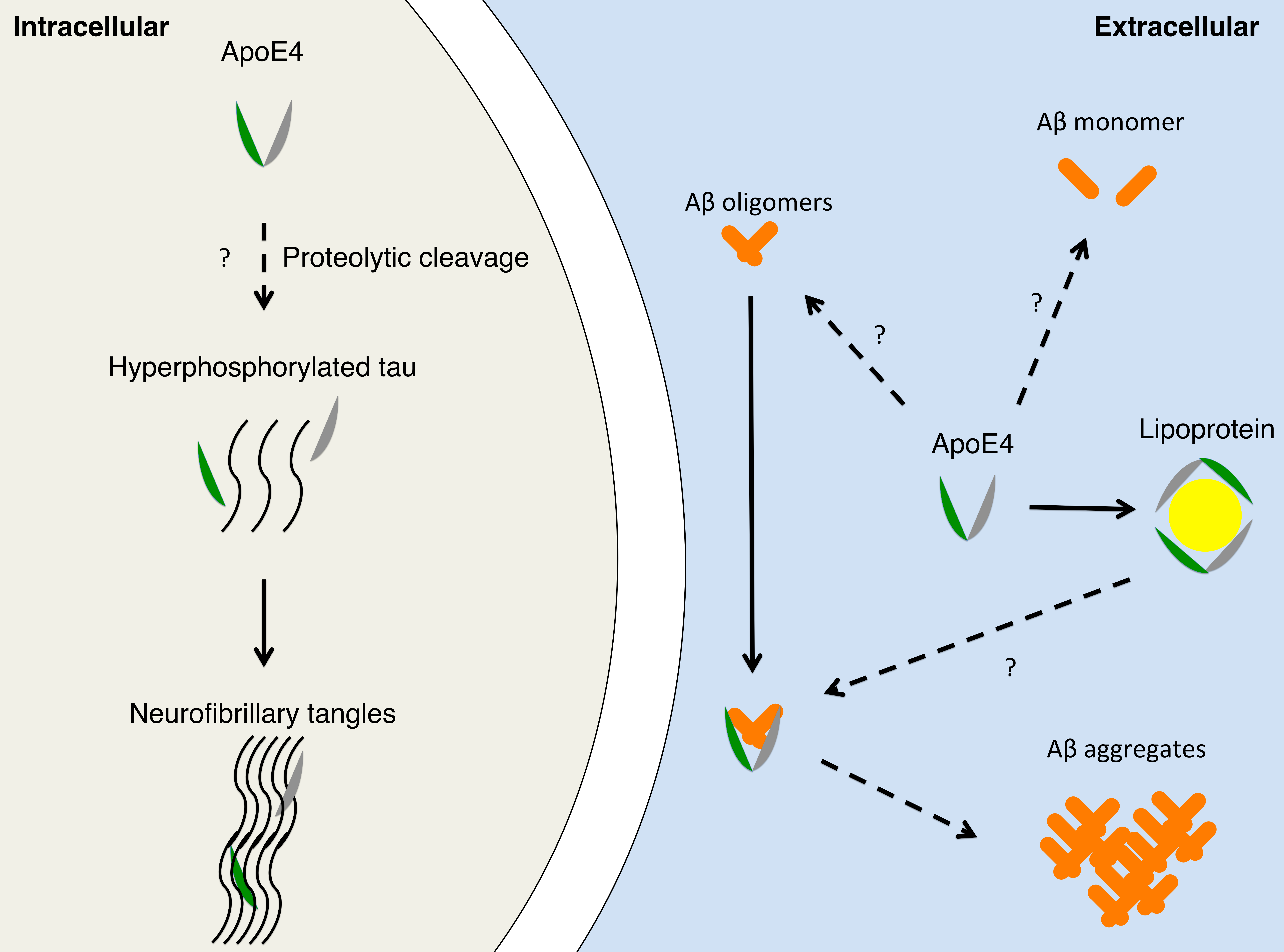

Supplement: S13 Fig — We speculate that the ApoE4 misfolded intermediate state may affect the kinetics of Aβ peptides aggregation and clearance. Concurrently, it may be favor the formation of intracellular neurofibrillary tangles. Further studies are required to elucidate the molecular mechanisms underlying the intracellular events leading to tau hyperphosphorylation and aggregation. Moreover, additional investigations are necessary to elucidate the molecular events at the basis of the interaction between ApoE4, lipids, and Aβ peptides. (TIF) [file pcbi.1004359.s013.tif]

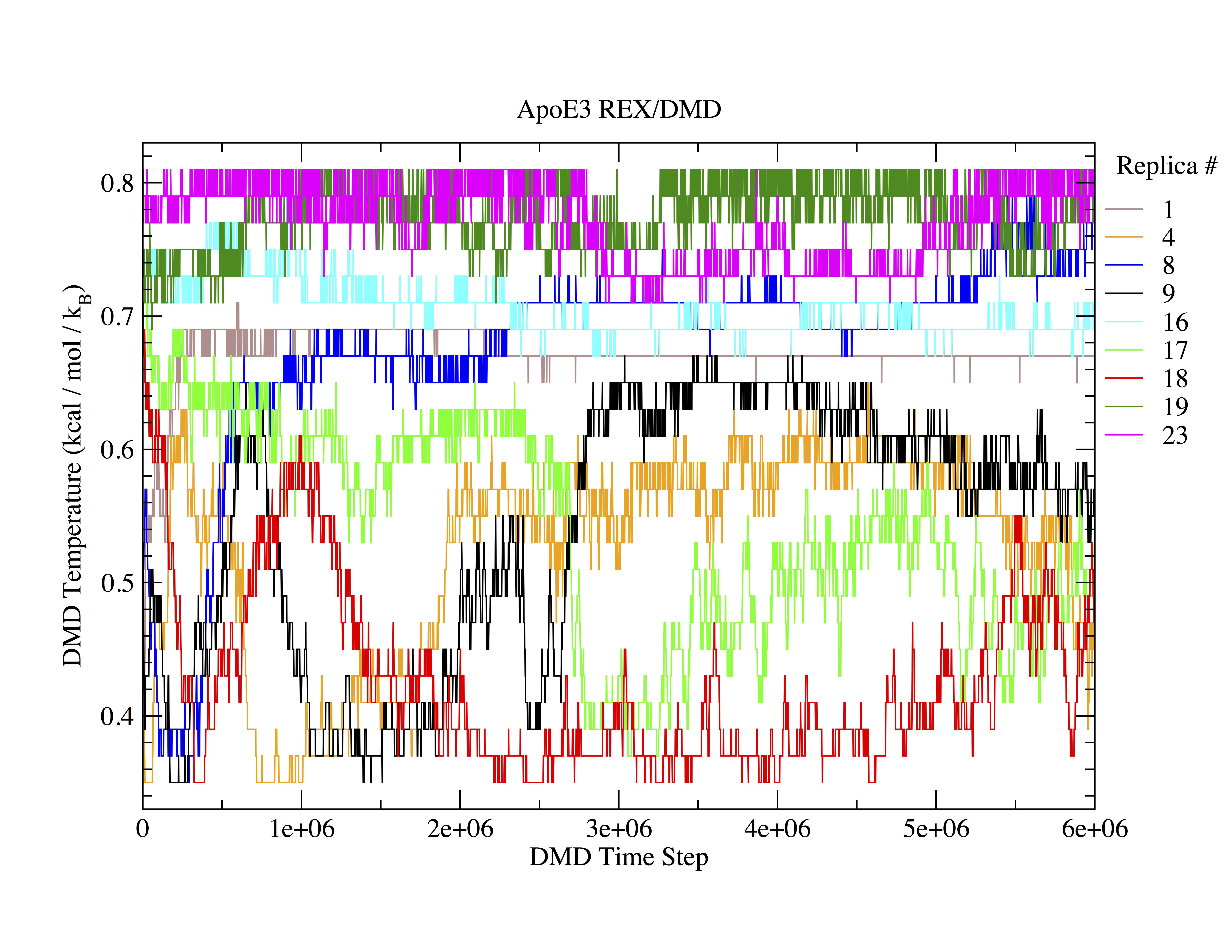

Supplement: S14 Fig — Representative replicas from our ApoE3 REX/DMD simulations are plots with their DMD temperature (kcal/mol/kB) as a function of time steps. There are replicas that travel through a wide range of temperature and those that are confined to a smaller spread in temperature space. (TIF) [file pcbi.1004359.s014.tif]

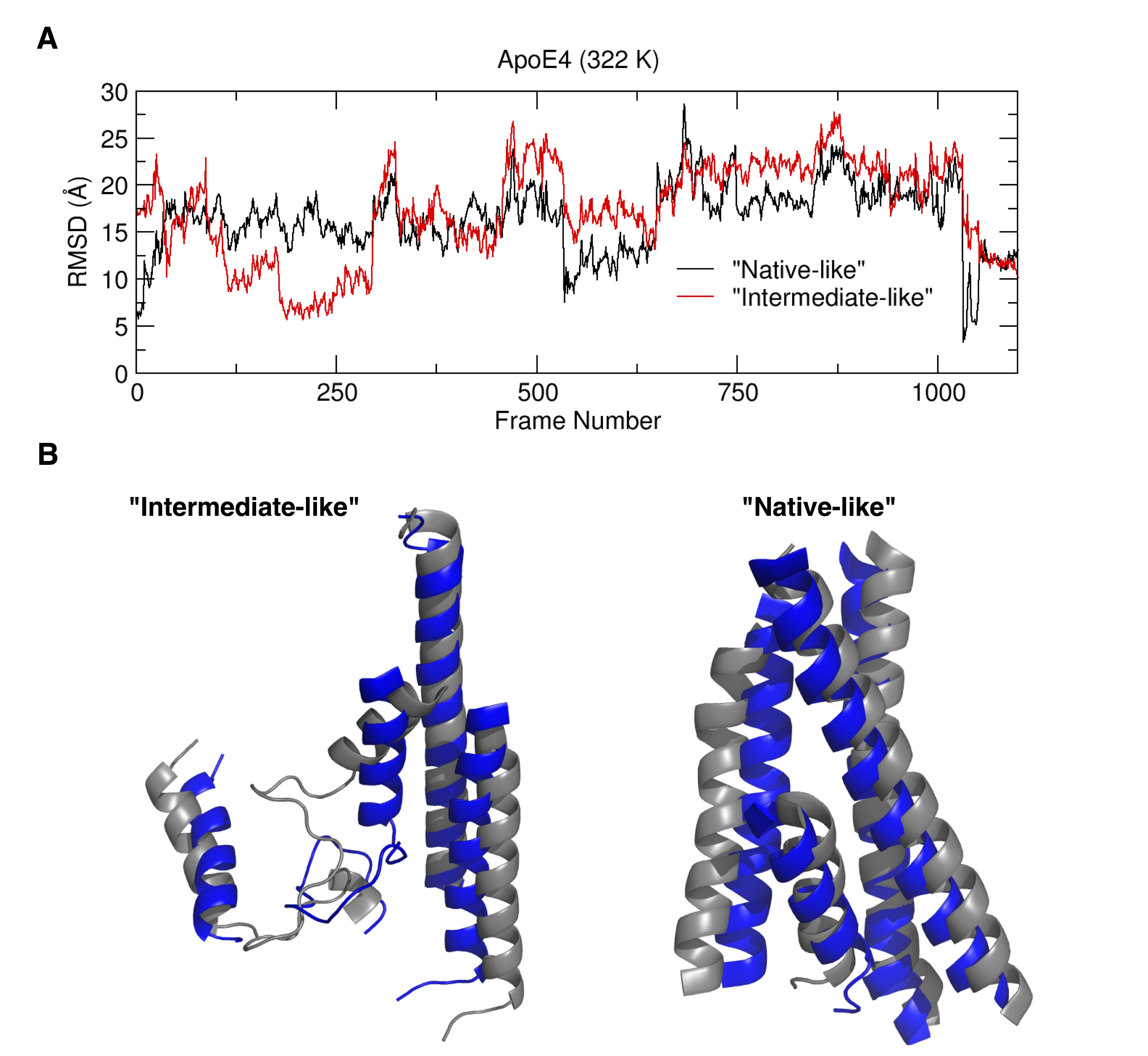

Supplement: S16 Fig — (A) The RMSD of the Cα atoms in the N-terminal domain helices was calculated between the trajectory of conformations at 322 K and the centroids found from clustering analysis at 309 K (T2) for the “native-like” state and at 328 K (T3) for the misfolded “intermediate-like” state. The RMSD values reveal that at the transition peak, ApoE4 visits both the “native-like” and misfolded “intermediate-like” N-terminal domain conformations (RMSD < 5 Å). (B) The conformations found at the transition state peak with the lowest RMSD values from (A) shown in blue are aligned with their corresponding centroid structures colored in gray. The “native-like” conformation has an RMSD value of 3.28 Å with its respective centroid while the “intermediate-like” conformation has an RMSD value of 5.68 Å suggesting that the same conformation is found in the free energy basin at 328 K. The RMSD for both (A) and (B) is calculated using the N-terminal domain helices because the flexibility of the C-terminal domain adds considerable noise as discussed in the Methods section and as shown in S3 Fig. (TIF) [file pcbi.1004359.s016.tif]

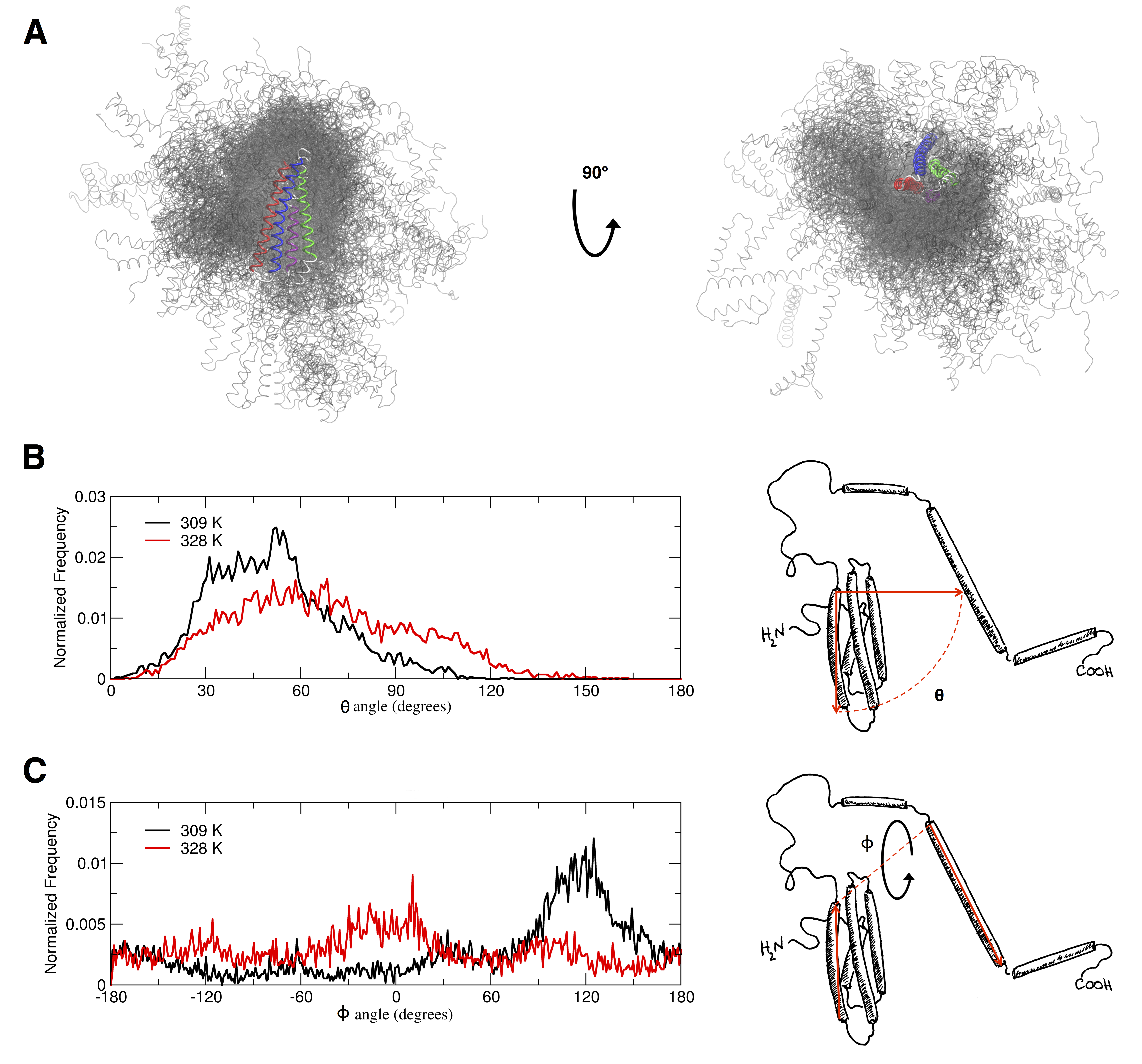

Supplement: S17 Fig — (A) The superposition of ApoE4 C-terminal conformations from REX/DMD simulations at 309 K with an aligned N-terminal domain reveals that the C-terminal domain explores a variety of positions relative to the N-terminal domain. The C-terminal domain assumes conformations next to the helix–1/helix–2 side of the N-terminal domain as suggested previously in literature as well as in conformations next to the helix–1/helix–4 and helix–4/helix–3 sides. The superposition of every 25th frame of C-terminal conformations from the REX/DMD trajectory is represented in grey. A representative conformation of the N-terminal domain alignment is shown with helix–1 (H1), helix–2 (H2), helix–3 (H3), and helix–4 (H4) in purple, green, blue, and red, cartoon, respectively. (B) The angle between the N-terminal domain and C-terminal domain shows the relative closeness between the two domains. An angle of zero degrees represents the N-terminal and C-terminal next to each other, while an angle of 180 degrees represents conformations with the C-terminal away from the sides of the N-terminal domain. The angle θ is measured using residues L148 and G165 in the most stable N-terminal helix, helix–4, and L252 representing the center of the most stable C-terminal domain helix. Note that the magnitude of the angle does not always correspond to a similar magnitude in distances between the two domains. (C) The dihedral angle φ between the N-terminal domain and the C-terminal domain shows the relative orientation between the two domains. An angle of zero degrees represents an anti-parallel orientation between the two domain helices while an angle of -180 or 180 degrees represents a parallel orientation. N-terminus to C-terminus is used for directionality. The angle φ is measured between vectors defined by the center of mass of residues E131 and G165 in the most stable N-terminal helix, helix–4, and residues E238 and F265 in the most stable C-terminal domain helix. The width of the histogram bins corresp [file pcbi.1004359.s017.tif]
