# Supplementary material for: ApoE4-specific Misfolded Intermediate Identified by Molecular Dynamics Simulations
Source: PLoS Comput Biol. 2015 Oct 27;11(10):e1004359. doi: 10.1371/journal.pcbi.1004359 (PMC4623519; doi:10.1371/journal.pcbi.1004359)
Supplement: S1 Table — (DOCX) [file pcbi.1004359.s018.docx]

**S1 Table. Human ApoE3 sequence.**

| KVEQAVETEPEPELRQQTEWQSGQRWELALGRFWDYLRWVQTLSEQVQEELLSSQVTQELRALMDETMKELKAYKSELEEQLTPVAEETRARLSKELQAAQARLGADMEDVCGRLVQYRGEVQAMLGQSTEELRVRLASHLRKLRKRLLRDADDLQKRLAVYQAGAREGAERGLSAIRERLGPLVEQGRVRAATVGSLAGQPLQERAQAWGERLRARMEEMGSRTRDRLDEVKEQVAEVRAKLEEQAQQIRLQAEAFQARLKSWFEPLVEDMQRQWAGLVEKVQAAVGTSAAPVPSDNH |
| --- |

The amino acid sequences of the three ApoE isoforms are identical with the exception of C112R and R158C mutations found in ApoE4 And ApoE2 variants, respectively.
