# Supplementary material for: ApoE4-specific Misfolded Intermediate Identified by Molecular Dynamics Simulations
Source: PLoS Comput Biol. 2015 Oct 27;11(10):e1004359. doi: 10.1371/journal.pcbi.1004359 (PMC4623519; doi:10.1371/journal.pcbi.1004359)
Supplement: S2 Table — (DOCX) [file pcbi.1004359.s019.docx]

Table S1. Structures found in our simulations have N-terminal helix conformations similar to solved crystal structures.

| **Ref. Protein (PDB ID)** | **RMSD** |
| --- | --- |
| ApoE2 (1LE2) [1] | 3.188 Å |
| ApoE3 (1NFN) [2] | 2.361 Å |
| ApoE3 (1OR3) [3] | 2.265 Å |
| ApoE3 (1OR2) [3] | 2.096 Å |
| ApoE3 (1LPE) [4] | 2.754 Å |
| ApoE3 (1BZ4) [3] | 2.714 Å |
| ApoE4 (1B68) [5] | 2.924 Å |
| ApoE4 (1GS9) [6] | 2.809 Å |
| ApoE4 (1LE4) [1] | 3.247 Å |

Root mean square deviation (RMSD) of the centroid the most populated clusters of ApoE isoforms’ conformations from local minima on the PMF-derived free energy landscapes at 275K. RMSD is computed over the Cα of the N-terminal domain helices. The C-terminal domain is not available in any of the ApoE crystal structures.
