# Supplementary material for: ApoE4-specific Misfolded Intermediate Identified by Molecular Dynamics Simulations
Source: PLoS Comput Biol. 2015 Oct 27;11(10):e1004359. doi: 10.1371/journal.pcbi.1004359 (PMC4623519; doi:10.1371/journal.pcbi.1004359)
Supplement: S3 Table — (DOCX) [file pcbi.1004359.s020.docx]

**S3 Table. RMSD between native-like ApoE conformations at 275 K**

| **ApoE Isoform** | E2 | E3 | E4 |
| --- | --- | --- | --- |
| E2 | 0 | 5.84 | 6.47 |
| E3 | 5.84 | 0 | 4.52 |
| E4 | 6.47 | 4.52 | 0 |

RMSD values in Å for the Cα atoms of the N-terminal helices for the centroids from clustering analysis at 275 K from REX/DMD simulations (Fig S2A-C).
