# Supplementary material for: ApoE4-specific Misfolded Intermediate Identified by Molecular Dynamics Simulations
Source: PLoS Comput Biol. 2015 Oct 27;11(10):e1004359. doi: 10.1371/journal.pcbi.1004359 (PMC4623519; doi:10.1371/journal.pcbi.1004359)
Supplement: S4 Table — (DOCX) [file pcbi.1004359.s021.docx]

**S4 Table. Population of ApoE intermediates at physiological temperatures.**

| **ApoE Isoform** | **Population of Intermediate from 300 to 310 K** |
| --- | --- |
| E2 | 70.66 % |
| E3 | 0.05 % |
| E4 | 11.12 % |

The populations of the ApoE intermediate states in the temperature range of 300 to 310 K were identified using RMSD against the centroid of the most populated cluster for intermediate state of each ApoE isoform (Fig 3BCD). Similarity was determined by an RMSD of the N-terminal domain helices with the centroids that is less than 15 Å.
