# Supplementary material for: ApoE4-specific Misfolded Intermediate Identified by Molecular Dynamics Simulations
Source: PLoS Comput Biol. 2015 Oct 27;11(10):e1004359. doi: 10.1371/journal.pcbi.1004359 (PMC4623519; doi:10.1371/journal.pcbi.1004359)
Supplement: S5 Table — (DOCX) [file pcbi.1004359.s022.docx]

**S5 Table. EPR distances in REX/DMD simulations.**

| **Frequency of distances at or below experimental measurements** | | | | | | |
| --- | --- | --- | --- | --- | --- | --- |
| **Residue Pairs** | **76-241** | **76-263** | **76-264** | **77-241** | **77-263** | **77-264** |
| **Distance Cutoff** | **(14.1 +/- 5 Å)** | **(21.2 +/- 5 Å)** | **(22 +/- 3Å)** | **(18.7 +/- 5 Å)** | **(22 +/- 3 Å)** | **(22 +/- 3 Å)** |
| **ApoE3 (318 K)** | 0.78% | 14.64% | 18.4% | 3.4% | 18.84% | 24.46% |
| **ApoE3 (338 K)** | 0.04% | 1.09% | 1.05% | 0.16% | 1.14% | 1.07% |
| **ApoE4 (309 K)** | 0.11% | 19.77% | 17.83% | 1.17% | 20.33% | 21.67% |
| **ApoE4 (328 K)** | 1.51% | 27.08% | 29.1% | 3.14% | 26.94% | 30.1% |
| **ApoE4 (328 K) Free Energy Basin** | 1.99% | 22.55% | 23.5% | 2.92% | 22.55% | 24.16% |

Six residue pairs within specific distance ranges were identified by Hatters et al. using electron paramagnetic resonance for 60% of ApoE4 tetramer conformations. Comparing these distance measurements with conformations extracted from our ApoE3 and ApoE4 monomer REX/DMD simulations reveals that we explore conformations that satisfy all six residue pairs with different frequencies. The low occupancy of experimental distance for residue pairs such as 76-241 and 77-241 may be explained by conformational differences between ApoE4 monomers and tetramers. In addition, at 338 K for ApoE3 and 328 K for ApoE4, corresponding to the temperature for their respective intermediate states, the distances are satisfied with a higher frequency in ApoE4 than in ApoE3 suggesting that these measurements may indeed be indicative of an ApoE4 intermediate conformation.
