# Supplementary material for: ApoE4-specific Misfolded Intermediate Identified by Molecular Dynamics Simulations
Source: PLoS Comput Biol. 2015 Oct 27;11(10):e1004359. doi: 10.1371/journal.pcbi.1004359 (PMC4623519; doi:10.1371/journal.pcbi.1004359)
Supplement: S6 Table — (DOCX) [file pcbi.1004359.s023.docx]

**S6 Table. Hydrophobic solvent exposed surface area of ApoE intermediate states**

| **ApoE Isoform** | **Average Hydrophobic Surface Area (1000 Å^2^)** |
| --- | --- |
| E2 | 10.13 +/- 0.74 |
| E3 | 10.47 +/- 0.52 |
| E4 | 8.63 +/- 0.72 |

The solvent exposed surface area was calculated for hydrophobic residues of the ApoE intermediate state clusters using a sphere with a 1.4 Å radius with Visual Molecular Dynamics software tools [7].
