# Supplementary material for: ApoE4-specific Misfolded Intermediate Identified by Molecular Dynamics Simulations
Source: PLoS Comput Biol. 2015 Oct 27;11(10):e1004359. doi: 10.1371/journal.pcbi.1004359 (PMC4623519; doi:10.1371/journal.pcbi.1004359)
Supplement: S7 Table — (DOCX) [file pcbi.1004359.s024.docx]

**S7 Table. ApoE structural and thermodynamic insights.** Summary of structural features and thermodynamic properties revealed by REX/DMD simulations for the three ApoE isoforms. ApoE4 exhibits multiple isoform-specific features related to the presence of a misfolded intermediate state.

| **Features** | **ApoE2** | **ApoE3** | **ApoE4** | **Reference** |
| --- | --- | --- | --- | --- |
| Mutation | C112/C158 | C112/R158 | R112/R158 | Table 1 |
| Hydrophobic core disruption* | 330 K | 331 K | 322 K | Fig 1 |
| Helix content at 275 K | 75.2 ± 3.7 % | 72.7 ± 2.6 % | 74.1 ± 3.8 % | S1 Fig |
| Beta strand content at 275 K | 2.2 ± 1.9 % | 3.6 ± 2.1 % | 2.9 ± 2.8 % | S1 Fig |
| Lowest RMSD with crystal structures | 3.2 Å  (PDB ID: 1LE2) | 2.1 Å  (PDB ID: 1OR2) | 2.8 Å  (PDB ID: 1GS9) | S2 Table |
| C-terminal domain range of motion at T3^¶^ | 28.2 ± 5.7 Å | 24.1 ± 6.3 Å | 30.9 ± 7.0 Å | S3 Fig |
| Distance between C-terminal segment  (residues 206-216) and helix-4 at T3^¶^ | 46.6 ± 13.5 Å | 55.4 ± 11.3 Å | 42.2 ± 12.7 Å | S11 Fig |
| Distance between C-terminal segment  (residues 247-257) and helix-4 at T3^¶^ | 62.9 ± 23.4 Å | 91.0 ± 15.5 Å | 42.9 ± 17.8 Å | S11 Fig |
| Hydrophobic surface area at T3^¶§^ | 10.1 ± 0.7 | 10.5 ± 0.5 | 8.6 ± 0.7 | S6 Table |
| Salt bridge distance (R61 – E255) at T3^¶^ | N/A | N/A | 52.9 ± 15.0 Å | S9 Fig |
| Average RMSD distribution of protein conformations in the most populated cluster at T3^¶^ | 16.3 ± 1.8 Å | 10.2 ± 1.5 Å | 16.8 ± 2.6 Å | S10 Fig |
| Population of intermediate state at 300 to 310 K | 70.7 % | 0.1 % | 11.1 % | S4 Table |

*First peak in the specific heat plot (Figure 1). ^¶^Analysis performed at temperature T3 (~340 K, ~338 K, and ~328 K for ApoE2, ApoE3 and ApoE4 respectively) correspond to the respective local minima of heat capacity curves for each ApoE isoform. ^§^Values expressed in the units of 1000 Å^2^.
