# Supplementary material for: ApoE4-specific Misfolded Intermediate Identified by Molecular Dynamics Simulations
Source: PLoS Comput Biol. 2015 Oct 27;11(10):e1004359. doi: 10.1371/journal.pcbi.1004359 (PMC4623519; doi:10.1371/journal.pcbi.1004359)
Supplement: S1 Text — (DOCX) [file pcbi.1004359.s025.docx]

**Supporting Information References**

1. Wilson C, Mau T, Weisgraber KH, Wardell MR, et al. (1994) Salt bridge relay triggers defective LDL receptor binding by a mutant apolipoprotein. Structure. 2: 713–8.

2. Dong LM, Parkin S, Trakhanov SD, Rupp B, Simmons T, et al. (1996) Novel mechanism for defective receptor binding of apolipoprotein E2 in type III hyperlipoproteinemia. Nat Struct Biol 3: 718–722.

3. Segelke BW, Forstner M, Knapp M, Trakhanov SD, Parkin S, et al. (2000) Conformational flexibility in the apolipoprotein E amino-terminal domain structure determined from three new crystal forms: implications for lipid binding. Protein Sci 9: 886–897. doi:10.1110/ps.9.5.886.

4. Wilson C, Wardell MR, Weisgraber KH, Mahley RW, Agard DA (1991) Three-dimensional structure of the LDL receptor-binding domain of human apolipoprotein E. Science 252: 1817–1822.

5. Dong J, Peters-Libeu CA, Weisgraber KH, Segelke BW, Rupp B, et al. (2001) Interaction of the N-terminal domain of apolipoprotein E4 with heparin. Biochemistry 40: 2826–2834. doi:10.1021/bi002417n.

6. Verderame JR, Kantardjieff K, Segelke BW, Weisgraber KH, Rupp B. (2003) Crystal Structure of the 22K Domain of Human Apolipoprotein E4. To be published. doi:10.2210/pdb1gs9/pdb.

7. Humphrey W, Dalke A, Schulten K. VMD: visual molecular dynamics. J Mol Graph. 1996;14: 33–8– 27–8.
